# Measuring Spatial Social Polarization in Public Health Research: A Scoping Review of Methods and Applications

**DOI:** 10.1007/s11524-024-00957-6

**Published:** 2025-03-10

**Authors:** Edwin M. McCulley, Lisa Frueh, Deiriai Myers, Samuel Jaros, Hoda S. Abdel Magid, Felicia Bayer, Gina S. Lovasi

**Affiliations:** 1 Urban Health Collaborative, Drexel University Dornsife School of Public Health, 3600 Market St, 7th Floor, Philadelphia, PA 19104 USA; 2 Department of Epidemiology and Biostatistics, Drexel University Dornsife School of Public Health, Philadelphia, PA USA; 3 Department of Environmental and Occupational Health, Drexel University Dornsife School of Public Health, Philadelphia, PA USA; 4 Department of Child and Adolescent Psychiatry & Behavioral Sciences, Children’s Hospital of Philadelphia, Philadelphia, PA USA; 5 Department of Epidemiology and Population Health, Stanford School of Medicine, Stanford, CA USA; 6 Department of Population and Public Health Sciences, University of Southern California, Los Angeles, CA USA

**Keywords:** Spatial social polarization, Public health, Scoping review, Health equity, Methodology, Social epidemiology, Spatial epidemiology

## Abstract

Spatial social polarization (SSP) refers to the uneven spatial distribution and subsequent concentration of polarized social and/or economic groups in a specified geographic area. However, there is heterogeneity in how SSP is measured and operationalized in research. To this end, we conducted a scoping review to characterize the use of SSP measures in public health research, providing a foundation for those seeking to navigate this complex literature, select measurement options, and identify opportunities for methodological development. Using a structured search strategy, we searched PubMed for any primary research, published since 2007, that examined the relationship between SSP and health outcomes. Across 117 included studies, we found a body of evidence that was primarily set in the United States (*n* = 104), published between 2020 and 2022 (*n* = 52), and focused on non-communicable diseases (*n* = 40). We found that defining SSP in the context of privilege, deprivation, and segregation returns a variety of measures. Among measures, we categorized 18 of them as SSP measures, with the Index of Concentration at the Extremes (*n* = 43) being the most common, and 5 of them as composite indices based on numerous underlying variables spanning several domains like education and race/ethnicity. While most employed a single SSP measure (*n* = 64), some included up to 5 measures to examine the robustness of findings or to identify how a multidimensional approach to SSP affected associations. Our findings fill a critical literature gap by summarizing options for operationalizing SSP measures and documenting their respective methodologies. Future research should consider using multiple SSP measures to capture the multidimensionality of SSP, widen the scope of health outcomes, and clearly explain the choice of measure(s) and methods used to derive them. Our findings can inform future research questions and help guide researchers in the selection and utilization of the various SSP measures.

**Supplementary Information:**

The online version contains supplementary material available at 10.1007/s11524-024-00957-6.

## Introduction

### Background

Across the globe, the COVID-19 pandemic catalyzed increased attention to health disparities research [1], this attention added urgency to calls to understand the spatial and social drivers of health disparities for other leading causes of death across populations. Recent research suggests that spatial social inequity, which includes economic and racial/ethnic polarization, operates at multiple levels [2] to affect population health outcomes [3]. Despite such efforts, there remain challenges in how spatial social polarization (SSP) is measured and operationalized in public health research.

Following decades of racial/ethnic and economic segregation in the United States (USA) [4–7], SSP measures have been increasingly used in US public health research to operationalize segregation as an exposure impacting population health [8]. Importantly, while SSP is not a US-only issue [9, 10], the vast majority of research on SSP has been US-based [11], and therefore much of our discussion focuses on US issues. Though it is increasingly common to employ SSP measures in public health research, particularly in the USA, the concept and terminology surrounding SSP, and its measurement have evolved over time. Conceptually, SSP is rooted in theories in the social sciences [12–14], that aim to explain the relational mechanisms by which spatial and social polarization co-occur. While the term SSP may not have a singular universally recognized originator, SSP terminology has been used in the fields of public health [15], geography [16], and sociology [17]. The evolution of the concepts and terminology related to SSP was concurrently marked by advances in SSP measurement.

### Defining Spatial Social Polarization

In order to define SSP, we must first define social polarization. Social polarization describes the division of a population into different groups with distinct social and/or economic characteristics which include or can be arrayed as between extremes of privilege and deprivation. Spatial social polarization refers to the uneven spatial distribution and subsequent concentration of polarized social and/or economic groups within a specified geographic area. However, there is little evidence available to guide the selection, utilization, and application of SSP measures.

### Development of Measures

The earliest attempt to categorize SSP measures was undertaken by American sociologists, Douglas Massey and Nancy Denton in 1988, with special focus on residential segregation [18]. Massey and Denton recognized that residential segregation was not a unidimensional construct and aimed to unpack its distinct dimensions (Table 1): concentration, evenness, exposure, clustering, and centralization. Concentration describes the relative physical space that is occupied by different groups [18], and evenness describes the spatial distribution of different group members in a given geography [18]. Exposure describes the degree of contact between members of different groups within a specified geography [18], and clustering describes the degree to which members of different groups cluster in space [18]. Last, centralization describes the location of different groups relative to the center of a geographic unit [18]. While these dimensions were developed in the context of residential segregation, we adapt them here to categorize measures of SSP more broadly.

**Table 1:** Dimensions of residential segregation for SSP measure classification

Dimension	Definition*
*Concentration*	The relative physical space occupied by different groups
*Evenness*	The spatial distribution of different group members within a unit
*Exposure*	The degree of contact between members of different groups within a unit
*Clustering*	The degree to which members of different groups cluster in space
*Centralization*	The location of different groups relative to the center of an urban area or other geographic unit

^*^Note: As defined by Massey DS and Denton NA. The dimensions of residential segregation. Social forces. 1988;67(2):281–315

### Implications for Public Health Research

While Massey and Denton provided a foundation for SSP measurement [18], their investigation is limited to residential segregation [19], which may not capture the interrelated dynamics of SSP. In order to examine how different social groups are spatially polarized, we must also consider relevant social systems and resources such as income, education, employment, and housing which are all spatially distributed [20]. This highlights a distinction between measures of residential segregation (which could refer to spatial separation of groups that are equivalent in access to social systems and resources) and measures of SSP. Moving beyond segregation measures, Feldman et al. [8] and Krieger et al. [3] were among the first to employ SSP measures in public health research and extended the measurement of SSP to several domains, including income and a combination of race/ethnicity and income.

This scoping review aims to characterize the use of SSP measures in recent public health literature, according to the dimensions described by Massey and Denton [18], providing a foundation for those seeking to navigate this complex literature, to select among measurement options, or to identify opportunities for further methodological development.

## Methods

### Information Sources and Eligibility Criteria

We conducted a scoping review of existing evidence to classify and characterize the measurement of SSP in public health research. We searched the National Center for Biotechnology Information database, PubMed, for primary research that employed any measure of SSP as an independent variable in a study of health outcomes among individuals or small area populations. Studies were eligible for inclusion if they met the following criteria: (1) written in English, (2) published between 2007 and 2022, (3) original research that included adjustments for individual-level characteristics, (4) characterized geographic areas smaller than cities or counties (e.g., areal units: neighborhoods, postal codes, and census tracts) with respect to polarization (e.g., to measure effects along a relative scale from deprivation to privilege) or segregation, (5) outcome was related to individual-level health and wellbeing, and (6) outcome was assessed concurrently with the characterization of the geographic area(s).

### Search Strategy

To identify relevant studies, we developed a structured search strategy based on search terms from the content of research articles by Feldman et al. 2015 [8], and Krieger et al. 2018 [3]. The structured search strategy was executed on January 2023, as follows: (“state” *OR* “county” *OR* “census tract” *OR* “geographic level”) *AND* (“spatial social polarization” *OR* “index of concentration at the extremes” *OR* “privilege” *OR* “deprivation” *OR* “dissimilarity” *OR* “segregation”). The search terms employed in this scoping review aimed to capture any measure of SSP among geographies smaller than cities or counties, regardless of the health outcome under study.

### Study Selection

After executing the search, references were compiled in EndNote, automatically screened for duplicates, and imported into Covidence, a web-based collaboration platform [21], for evidence screening and synthesis. References were subjected to independent abstract screening by two members of the research team (i.e., EMM and DM) based on the eligibility criteria, with disputes resolved via consensus by a third member (i.e., HSAM) of the research team. Following the abstract screening, we accessed the full-text versions of eligible references and proceeded with the data abstraction process.

### Data Abstraction Process and Data Items

Eligible references were abstracted to assemble information on publication year, study design, study setting, study population, sample size, health outcome(s), and characteristics of SSP measures including SSP measure name, SSP measure formula (if available), and related domain(s). Information collected during the data abstraction process was independently documented and verified by another member of the research team.

### Methods of Analysis and Synthesis of Results

Following screening, selection, and data abstraction, results were synthesized based on relevant study attributes and SSP measure characteristics. First, studies were grouped according to the data items described above. Health outcomes were classified, using the methods described by Henson et al. 2020 [22], as either: non-communicable diseases, communicable diseases, mortality, general physical health, maternal and perinatal health, injuries, general mental health, or quality of life. Then, we systematically characterized each SSP measure according to the dimensions described by Massey and Denton [18] (Table 1), and compiled a list of unique SSP measures employed across the body of literature. A summarized description of each measure included the following: the measure formula, applicable domains (e.g., race/ethnicity, income, education), and accompanying references. Finally, we tabulated results for presentation as guided by the Preferred Reporting Items for Systematic Review and Meta-Analysis Extension for Scoping Reviews (PRISMA ScR) [23, 24] and provided a narrative synthesis. In this scoping review, we adopted a flexible approach to synthesis using scoping review methodology [25] rather than adhering to a predefined study protocol.

## Results

### Search and Study Selection

**Fig. 1:**
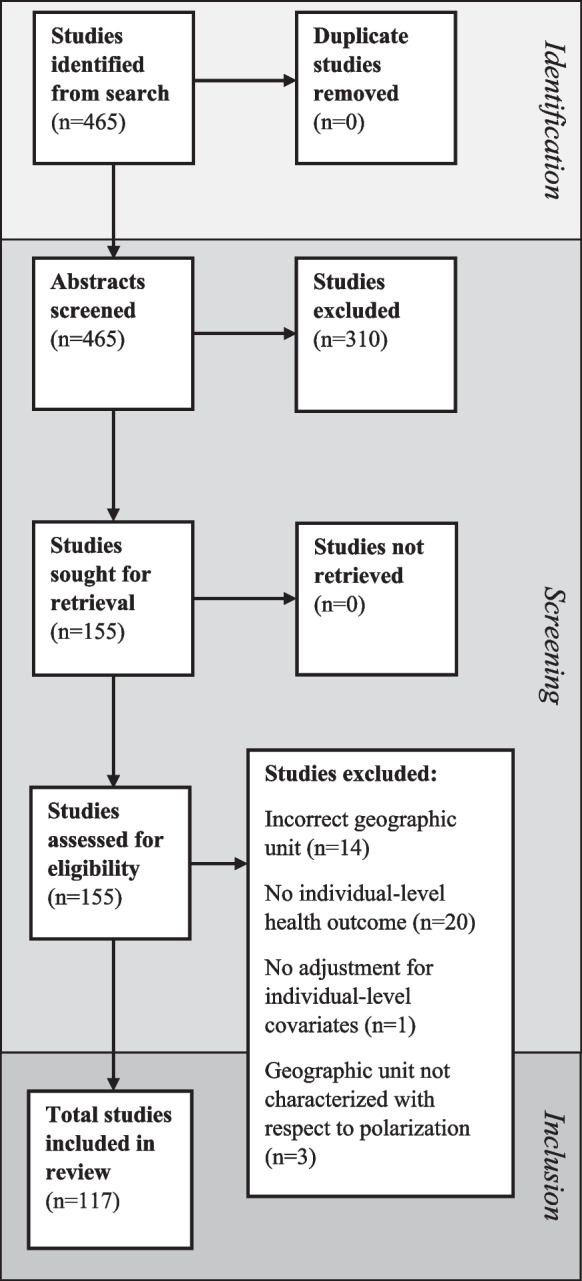
PRISMA flowchart

Our primary search initially identified 465 articles (Fig. 1). We excluded 310 articles based on title/abstract review. We retrieved 155 full-text articles for eligibility assessment, leading to the exclusion of 38 articles. Reasons for exclusion included geographic measurement scale (i.e., no geographic units considered smaller than cities or counties), no individual-level health outcome, no measurement of polarization or segregation, and not original research. Post-eligibility assessment, we included 117 articles in the review.

### Overview of Studies

The characteristics of included articles (*n* = 117) are presented in Table 2. We identified a wide body of literature published between 2007 and 2022 that indicates a clear trend by publication year; 34.2% (*n* = 40) was published between 2015 and 2019, and nearly 45% was published (*n* = 52) from 2020 to 2022. A majority of the studies were set in the USA (*n* = 104), followed by Canada (*n* = 10). Among included articles, the median sample size was 21,403 with an interquartile range of 144,673 (Q1: 2678, Q3: 147,351). Across included articles, nearly 46% of the evidence employed a cohort design (*n* = 54), and 42% employed a cross-sectional design (*n* = 49). The number of SSP measures used in each study varied, 54.7% (*n* = 64) utilized 1 SSP measure, 36.8% (*n* = 43) utilized between 2 and 3 different SSP measures, and 8.5% (*n* = 10) utilized 4 to 5 different SSP measures. As for health outcomes, most studies focused on non-communicable diseases (*n* = 40) followed by mortality (*n* = 27), general physical health (*n* = 16), maternal/perinatal health (*n* = 15), injuries (*n* = 7), communicable diseases (*n* = 6), general mental health (*n* = 3), and quality of life (*n* = 3).

**Table 2:** Characteristics of included studies

**Total** (*n*, *n*%)	117	100%
**Publication year** (*n*, *n*%)		
2007 to 2009	9	7.7%
2010 to 2014	16	13.7%
2015 to 2019	40	34.2%
2020 to 2022	52	44.4%
**Country** (*n*, *n*%)		
United States	104	88.8%
Canada	10	8.5%
Italy	1	0.9%
France	1	0.9%
India	1	0.9%
**Study design** (*n*, *n*%)		
Cohort	54	46.2%
Cross-sectional	49	41.9%
Other	10	8.5%
Case–control	2	1.7%
RCT	2	1.7%
**Health outcomes** (*n*, *n*%)		
Non-communicable diseases	40	34.2%
Mortality	27	23.1%
General physical health	16	13.6%
Maternal and perinatal health	15	12.8%
Injuries	7	6.0%
Communicable diseases	6	5.1%
General mental health	3	2.6%
Quality of life	3	2.6%
**Sample size** (mean, SD)		
	3,997,718	30,477,369
**Number of SSP measures employed** (*n*, *n*%)		
1 exposure measure	64	54.7%
2–3 exposure measures	43	36.8%
4–5 exposure measures	10	8.5%

### Measurement Classification

Of the 23 measures identified by our review, 18 were SSP measures and 5 were composite indices. SSP measures that were clearly defined were classified according to the dimensions of residential segregation described by Massey and Denton [18], and are presented in Table 3; which describes each of the 18 SSP measures, and provides a brief background, the measure formula, formula details, annotated strengths and limitations, plus relevant domains. While some measures are exclusively used for SSP research (e.g., Index of Concentration at the Extremes [ICE]), others are measures that are not specific to SSP research, though are utilized in this context in the research presented here (e.g., Getis-Ord G* and relative ratios).

**Table 3:** Spatial social polarization (SSP) measure details

Name	Background	Formula	Formula details	Strengths and limitations	Dimension	Domain	References
**Index of Concentration at the Extremes** (ICE)	ICE was initially developed to measure economic polarization in both privileged and deprived groups simultaneously using 1 measure [19]. It was extended in 2015 to capture both racial and economic disparities [26]. ICE is a continuous measure that ranges from −1 to 1, negative values indicate concentrated deprivation and positive values indicate concentrated privilege	${ICE}_{i}= \frac{({A}_{i}-{P}_{i})}{{T}_{i}}$	A_i_ = Number of privileged persons in areal unit i (e.g., in 80th income percentile) P_i_ = Number of deprived persons in areal unit i (e.g., in 20th income percentile) T_i_ = Total population with known income level in areal unit i	**STRENGTHS:** Single metric avoids issues of multicollinearity that are common when separate measures of privilege and deprivation are used **LIMITATIONS:** Choice of distribution cutoff points affects classification as either privileged or disadvantaged	*Concentration*	*Income*	[3, 8, 15, 26–54]
						*Race*	[3, 8, 15, 26, 27, 29–39, 41, 42, 45, 55, 46, 47, 50–54, 135–138]
						*Race/income*	[3, 8, 15, 26, 27, 30–37, 41–43, 45–47, 50–52, 56–60]
						*Education*	[8, 26, 53]
						*Language*	[53, 61]
						*Nativity*	[53]
						*Home ownership*	[36]
**Index of Dissimilarity** *(Dissimilarity Index, Duncan Index of Dissimilarity)*	The dissimilarity index (D) was initially developed in 1955 to measure occupational segregation [62], and adapted to measure residential segregation [18]. It represents the proportion of a group that would need to move to achieve a uniform population distribution. The minimum value of 0 corresponds to no dissimilarity, and the maximum of 1 corresponds to complete segregation or separation. This index can be adapted to spatial data using the spatial dissimilarity index [63]	$D= \frac{1}{2} \sum \limits_{i=1}^{n}|\frac{{w}_{i}}{{W}_{T}}-\frac{{b}_{i}}{{B}_{T}}|$	*n* = Total number of areal units in the study area w_i_ = Number of individuals in group w, in areal unit i W_T_ = Total number of group w individuals in the study area b_i_ = Number of individuals in group b, in areal unit i B_T_ = Total number of group b individuals in the study area	**STRENGTHS:** Easy to compute, and easy to interpret. Can be used to compare overall segregation levels within a geography over time, or between different geographies **LIMITATIONS:** Binary measure only captures segregation that exists between two groups at a single point in time. Does not provide a single value for all groups, which may conceal segregation among other (2 + groups). D becomes increasingly unstable as the overall population, or populations of comparison groups decreases. Aspatial index does not consider the proximity of various areal units	*Evenness*	*Race*	[42, 55, 64–73, 89, 139–146]
						*Income*	[74]
**Isolation Index**	The isolation index was initially proposed by Bell [75], refined by Lieberson [76], and formally defined by Massey [18], to measure the extent to which minority group members are exposed to other minority group members, using a weighted average for each unit’s minority proportion. Index ranges from 0 to 1 and represents the probability that a minority group member shares a unit with another minority group member [77]	$I=\sum \limits_{i=1}^{n}[\left(\frac{{x}_{i}}{X}\right)\left(\frac{{x}_{i}}{{t}_{i}}\right)]$	*n* = The number of areal units in the study area, ordered smallest to largest by land area x_i_ = The minority population of areal unit i X = The sum of all x_i_ (i.e., the total minority population) t_i_ = The total population of areal unit i	**STRENGTHS:** Measures the relative dissimilarity of the distribution, regardless of any difference in group size. Can remove asymmetry by using methods like the “Local Krivo Isolation Index” or “Correlation Index” **LIMITATIONS:** Asymmetry when 2 groups have the same proportion in the population. Earlier formulations rely on absolute size of groups [75, 76]	*Exposure*	*Race*	[32, 45, 65, 69, 71, 73, 78–91]
						*Income*	[74]
**Relative Ratios**	Relative ratios are a method to measure relative concentration, which denotes measures of frequency to provide a relative comparison between groups. Values range from -∞ to ∞, and interpretation varies according to the choice of comparison/referent group	${RR}_{i}= \frac{{A}_{i}}{{B}_{i}}$	A_i_ = The measure of frequency in the deprived (i.e., comparison) group, for areal unit i B_i_ = The measure of frequency in the privileged (i.e., referent) group, for areal unit i	**STRENGTHS:** Simplistic yet flexible measure of population composition **LIMITATIONS:** Employing 2 or more comparison groups necessitates multiple comparisons	*Concentration*	*Race*	[92–96, 147, 148, 149]
						*Income*	[44, 92, 96]
**Gini Coefficient** *(Gini Index)*	The Gini coefficient (G) was developed by Corrado Gini in 1912, as a relative measure of income inequality [97]. The Gini coefficient is a measure of inequality among particular values of a frequency distribution. A coefficient of 0 indicates perfect equality, and 1 indicates perfect inequality	$G=1- \sum \limits_{i=1}^{n}{{(p}_{i})}^{2}$ $Gb=1-\sum \limits_{i=0}^{n-1}({Y}_{i+1}+{Y}_{i})({X}_{i+1}-{X}_{i})$	*n* = The total number of areal units p_i_ = The probability of an areal unit being assigned a particular value of a distribution Y_i_ = The cumulative proportion of the variable in group i X_i_ = The cumulative proportion of the population in group i	**STRENGTHS:** Single summary statistic allows for the examination of inequities in 1 variable, or 2 variables using Browns Formula (Gb) [98] **LIMITATIONS:** G alone cannot differentiate between different types of inequities. The characteristics of Gb are not well documented	*Evenness*	*Race*	[45, 99]
						*Income*	[72, 78]
**Krivo Local Isolation Index**	The Krivo Local Isolation Index was developed to measure segregation [100]. It measures the areal unit-level probability of interaction between individuals belonging to 2 different groups compared with what would be expected in the entire study area (e.g., city) if residents were not spatially clustered. Positive values indicate a proportionate decrease in the chance of interaction between groups X and Y compared with the probability of random interaction in the study area [101]. Negative values indicate greater exposure among groups	${LS}_{i*xy}=1- \frac{{~}^{(\sum \nolimits_{j}{c}_{ij}{y}_{ij})}\!\left/ \!{~}_{(\sum \nolimits_{j}{c}_{ij}({x}_{j}{y}_{j}))}\right.}{{~}^{(\sum \nolimits_{j}{y}_{j})}\!\left/ \!{~}_{(\sum \nolimits_{j}\left({x}_{j}+{y}_{j}\right))}\right.}$	x = The number of people in group X that live in areal units i and j y = The number of people in group Y that live in areal units i and j c_ij_ = The value of cell ij in a spatial weights’ matrix, which equals 1 if areal units i and j share a border or if i = j, and 0 otherwise	**STRENGTHS:** Provides a localized measure of the probability of interaction between individuals in two groups within specific areal units. By considering a spatial weights matrix that accounts for shared borders, the index incorporates the spatial context of areal units. Accounts for the asymmetry inherent to the “Isolation Index” **LIMITATIONS:** Limited to the comparison of 2 groups, and results may be sensitive to the chosen spatial scale of analysis	*Exposure*	*Race*	[101]
						*Income*	[101]
Local Getis Ord G* statistic	G* statistics are z-scores that assess how different the racial composition of a particular areal unit and its neighboring units are, compared to the mean racial composition of the larger study area (e.g., city). The z-scores and accompanying p-values tell us where units with high or low values spatially cluster, with larger z-scores indicating greater spatial clustering of high values, and smaller z-scores indicating greater spatial clustering of low values. A p-value will be statistically significant if an areal unit with high values is surrounded by other units with high values [102]	${G}_{i}^{*}= \frac{\sum \nolimits_{j=1}^{n}{w}_{ij}{x}_{j}-\overline{X }\sum \nolimits_{j=1}^{n}{w}_{ij}}{S\sqrt{\frac{[n\sum \nolimits_{j=1}^{n}{w}_{ij}^{2}{-(\sum \nolimits_{j=1}^{n}{w}_{ij})}^{2}]}{n-1}}}$ $where$ $\overline{X }= \frac{\sum \nolimits_{j=1}^{n}{x}_{j}}{n}$ $S=\sqrt{\frac{\sum \nolimits_{j=1}^{n}{x}_{j}^{2}}{n}-{(\overline{X })}^{2}}$	x_j_ = Attribute value (e.g., % population belonging to a certain social group) for areal unit j w_ij_ = The spatial weight between areal units i and j n = Total number of areal units	**STRENGTHS:** Useful for hot spot analyses. Can adapt to larger geographies and assess clustering by using the Global Getis Ord G* statistic **LIMITATIONS:** There exists a trade-off between the accuracy of the G* statistic and the precision of the search radius, reducing the search radius tends to reduce the number of areas with significant p-values	*Clustering*	*Race*	[103–108]
**Location Quotient**	Location quotient measures the relative concentration (i.e., proportion), of minority group members in the study area (e.g., city) compared to the proportion in a particular areal unit (e.g., census tract) [109]. Location quotient ranges from 0 to ∞; lesser values indicate that the proportion of minority group members in the smaller areal unit is less than the proportion of the same group in the entire study area. Larger values indicate that the proportion of minority group members in the areal unit is greater than the proportion of the same group in the entire study area	${LQ}_{im}= \frac{(\frac{{x}_{im}}{{X}_{i}})}{(\frac{{Y}_{m}}{Y})}$	LQ_im_ = The value of the i^th^ areal unit in a study area for minority group m x_im_ = The number of individuals from minority group m living in the i^th^ areal unit X_i_ = Total number of residents in the i^th^ areal unit Y_m_ = Total number of individuals from minority group m in the study area Y = Total number of residents in the study area	**STRENGTHS:** Measure compares 2 areal units and allows us to examine relative deprivation **LIMITATIONS:** LQ_im_ is sensitive to small-area estimation therefore diminished population sizes may bias the magnitude of LQ_im_	*Concentration*	*Race*	[109–111]
**Redlining Index**	A measure of the relative disparity in mortgage loan denial between 2 groups. The redlining index uses a pooled odds ratio (OR) to place each area along a continuum of disparities in mortgage loan denial. Interpretation follows that of pooled ORs, such as those estimated by the Mantel–Haenszel method. This is not to be confused with “historical redlining scores”, which rely on historical HOLC grades and grade weights	${\overline{OR} }_{loan\;denial}= \frac{\sum \nolimits_{i=1}^{k}(\frac{{a}_{i}{d}_{i}}{{n}_{i}})}{\sum \nolimits_{i=1}^{k}(\frac{{b}_{i}{c}_{i}}{{n}_{i}})}$	a_i_ = The number of individuals in group 1 that had approved loans d_i_ = The number of individuals in group 2 that had denied loans b_i_ = The number of individuals in group 1 that had denied loans c_i_ = The number of individuals in group 2 that had approved loans n_i_ = The total number of individuals in groups 1 and 2	**STRENGTHS:** Can apply stratified models or random effects models to account for multilevel geographies **LIMITATIONS:** Offers limited insight on historical redlining, as HOLC grades are not involved in calculating the redlining index. Due to the non-collapsibility of the OR, the estimates of the conditional OR may differ from the marginal OR	*Concentration*	*Race*	[64, 112]
**Entropy** *(Information Index)*	The entropy index was developed as a measure of segregation by Theil & Finizza [113]. Entropy describes the extent of a study area’s racial/ethnic diversity and reaches it maximum when each racial/ethnic group is equally represented in the study area [77]. The entropy index provides a weighted measure of each areal unit’s departure from the study area’s entropy. The entropy index varies between 0 and 1; a value of 0 corresponds to all areal units having the same composition as the city, and a value of 1 corresponds to all units being composed of only 1 group	$H= \frac{\sum \nolimits_{i=1}^{n}{t}_{i}(E-{E}_{i})}{ET}$ $where$ $E=Pln\left(\frac{1}{P}\right)+\left(1-P\right)\text{ln}(\frac{1}{1-P})$ ${E}_{i}={p}_{i}ln\left(\frac{1}{{p}_{i}}\right)+\left(1-{p}_{i}\right)\text{ln}(\frac{1}{1-{p}_{i}})$	P = Proportion of minority group members to total population in a study area P_i_ = Proportion of minority group members to total population in areal unit i T = Total population of majority group members and minority group members in the study area t_i_ = Total population of majority group members and minority group members in areal unit i	**STRENGTHS:** Measures the spatial distribution of multiple groups simultaneously, while capturing racial/ethnic diversity **LIMITATIONS:** Scale dependent and sensitive to outliers, meaning that extreme values may significantly impact the index	*Evenness*	*Race*	[65, 79]
**Delta** *(Delta Index)*	The Delta index was founded by Hoover [114], and adapted to measure spatial concentration [115]. The Delta index calculates the percentage of minority members living in an areal unit with an above-average concentration of minority residents. The maximum and minimum values of the delta index are 1 and 0, indicating the proportion of a minority group that would have to move in order to achieve a uniform density across units [77]	$Delta=0.5\sum \limits_{i=1}^{n}|\frac{{x}_{i}}{X}-\frac{{a}_{i}}{A}|$	*n* = The number of areal units in the study area, ordered smallest to largest x_i_ = The minority population of areal unit i X = The sum of all x_i_ (i.e., total minority population in the study area) a_i_ = The land area of areal unit i A = The sum of all a_i_ (i.e., total land area in the study area)	**STRENGTHS:** Easy to compute and interpret **LIMITATIONS:** Requires information on the land area occupied by each unit	*Concentration*	*Race*	[78, 79]
**Spatial Proximity Index** *(Index of Spatial Proximity)*	The Spatial Proximity index (SP) was first proposed by White [17], as an index to measure the spatial clustering of groups. The index corresponds to the average intra-group proximities, weighted by the proportion of each group in the population [77]. SP = 1 corresponds to no differential clustering between majority (Y) and minority (X) group members, SP > 1 indicates that members of each group live closer to one another than to members of the other group (e.g., more clustering), and SP < 1 indicates that members of both groups live closer to each other than to members of their own group (e.g., less clustering)	$SP= \frac{\left({XP}_{xx}+{YP}_{yy}\right)}{{TP}_{tt}}$ $where$ ${P}_{gg}=\sum \limits_{i=1}^{n}\sum \limits_{j=1}^{n}\frac{({g}_{i}{g}_{j}{c}_{ij})}{{G}^{2}}$ *(g, G)* = *(x, X), (y, Y), (t, T)* $Relative\;Clustering= \frac{{P}_{xx}}{{P}_{yy}}-1$	x_i_, x_j_ = The minority population of areal units i or j X = The total minority population of the study area y_i_, y_j_ = The majority population of areal units i or j Y = The total majority population of the study area t_i_, t_j_ = The total population of areal units i or j T = The total population in the study area P_xx_ = The average proximity between minority group members P_yy_ = The average proximity between majority group members P_tt_ = The average proximity among all members of the population c_ij_ = exp(-d_ij_), Transformation of the distance between centroids of areal units i and j (d_ij_)	**STRENGTHS:** Spatial index considers the proximity of various neighborhoods. Can derive a measure of “Relative Clustering” by subtracting one so that index ranges from 0 to 1.0, with higher values reflecting higher segregation [18] **LIMITATIONS:** Application is limited to situations where the spatial arrangement of population groups is a key concern. Index may be sensitive to the chosen geographic scale of analysis	*Clustering*	*Race*	[73, 79]
**Exposure Index** *(Interaction Index)*	The Exposure Index, or Interaction Index was defined by Lieberson in 1981 [116], as the probability that a randomly drawn member of the minority group shares an area with a member of the majority group. The exposure index measures the extent to which minority group members are exposed to majority group members. The exposure index ranges from 0 to 1, lower values indicate greater segregation among groups and higher values indicate less segregation among groups [18]. The interaction index can complement the “Isolation Index”, in a 2-group comparison the Isolation and Interaction Indices sum to 1; lower values of interaction and higher values of isolation are both indicative of greater segregation	$x{P}^{*}y= \sum \limits_{i=1}^{n}[(\frac{{x}_{i}}{X})(\frac{{y}_{i}}{{t}_{i}})]$	*n* = The number of areal units (e.g., census tracts) in the study area, ordered smallest to largest by land area x_i_ = The minority population of areal unit i X = The sum of all x_i_ (total minority population in the study area) y_i_ = The majority population of areal unit i t_i_ = The total population of areal unit i	**STRENGTHS:** When employed with the “Isolation Index”, it can be used to assess segregation in terms of overall residential exposure. Allows for the comparison of inter- and intra-group probabilities **LIMITATIONS:** Binary focus may oversimplify the complexity of multi-group interactions such as those found in diverse populations. Scale dependent index does not capture the spatial arrangement of groups within an area and depends on the relative sizes of groups being compared (i.e., asymmetric)	*Exposure*	*Race*	[64]
**Kernel Density Estimation**	Kernel Density Estimation (KDE) is used to summarize the spatial distribution and clustering of a population. KDE methods have been extended to accommodate spatial data, which can be used to measure residential segregation. Relative composition and subsequent clustering are measured by multiplying sample data by a probability density function that is continuously applied across the data, then summing results to yield a single estimate of the underlying probability density function [117]. Resulting KDE estimates can be divided by the total population and multiplied by 100 to provide the percent of group members at each population-weighted centroid	$y\left(x\right)= \frac{1}{n}\sum \limits_{i=1}^{n}K(\frac{x-{X}_{i}}{h})$ $$K\left(r\right)=\begin{array}{c}c\left(h\right){\left(1-{\left(\frac{r}{h}\right)}^{2}\right)}^{2}\\\end{array}$$ $where$ *r* < = *h, 0 otherwise * $c\left(h\right)= \frac{3}{\pi {h}^{2}}$	y = The estimated probability density function X_i_ = The minority population of areal unit i x = The sum of all X_i_ (i.e., total minority population in the study area) K = The kernel function; spatially, it can be expressed as a function of r r = Distance from kernels central point h = Kernel bandwidth c(h) = Scaling factor to ensure function sums to unity	**STRENGTHS:** Provides better estimation of a population’s spatial distribution beyond histograms. Population data for neighboring census tracts are incorporated in measuring an areal unit’s racial residential segregation. Provides a smooth and continuous representation of the underlying probability density function **LIMITATIONS:** Tends to over smooth near the boundaries of the data, leading to underestimation of density near the edges of the observed range; known as the boundary effect. KDE can be computationally intensive. Choice of bandwidth is often subjective, and different bandwidths may lead to different interpretations of the data	*Clustering*	*Race*	[118]
**Atkinson Index**	The Atkinson Index was designed to evaluate segregation and income inequality [119]. The Atkinson Index allows for the differential weighting of units at different points along the distribution; weights are decided by the researcher using the shape parameter and correspond to areas of where minority group members are either over- or under-represented. Larger values of the shape parameter (b: 0.5–1) indicate areas where the proportion of minorities is greater than the study area average (e.g., over-representation); smaller values (b: 0–0.5) indicate units where the proportion of minorities is smaller than the study area average (e.g., under-representation) [77]. A shape parameter of 0.5 indicates that both areas contribute equally. Both b & AI range from 0 to 1; 0 for minimal segregation, 1 for maximum segregation [77]	$AI=1-(\frac{P}{1-P})|\frac{1}{PT}\sum \limits_{i=1}^{n}{{[(1-{p}_{i})}^{(1-b)}{p}_{i}^{b}{t}_{i}]|}^{(\frac{1}{1-b})}$	P = The ratio of minority group members (X) to the total population (T) t_i_ = The total population of areal unit i T = The total population in the study area (i.e., sum of all t_i_) p_i_ = The ratio of minority group members (x_i_) to the total population in areal unit i (t_i_) b = The shape parameter	**STRENGTHS:** The Atkinson Index allows for the incorporation of different levels of inequality aversion through the shape parameter. Different values of b lead to different Atkinson Indices, providing a range of inequality measures. It is size invariant; the measure is unchanged when the number of individuals in each group is multiplied by a constant **LIMITATIONS:** Subjective selection of shape parameter can impact comparability. The index can be relatively insensitive to changes in the middle- range and instead focuses more on the distribution extremes	*Evenness*	*Income*	[80]
**Absolute Centralization Index**	The Absolute centralization index (ACE) captures centralization, it ranges from −1 to 1 with > 0 values indicating that minority group members reside closer to the city center, and < 0 values indicating that minority group members reside outside of the city center; a value of 0 indicates that the minority group has a uniform distribution throughout the city [18]. ACE can be extended to consider a majority and minority group using the “Relative Centralization Index” (RCE) [18], which describes the share of minority group that would have to change residential areas to match the relative centralization of the majority group; positive values indicate that minority groups live closer to the center relative to the majority group, and vice-versa	$ACE= \sum \limits_{i=1}^{m}({X}_{i-1}{A}_{i})-\sum \limits_{i=1}^{m}({X}_{i}{A}_{i-1})$ $RCE=\sum \limits_{i=1}^{m}({X}_{i-1}{Y}_{i})-\sum \limits_{i=1}^{m}({X}_{i}{Y}_{i-1})$	X_i_ = The minority population of areal unit i A_i_ = The land area of areal unit i Y_i_ = The majority population of areal unit i M = The number of areal units, ordered by increasing distance from city center	**STRENGTHS:** Straightforward spatial interpretation. RCE can complement ACE **LIMITATIONS:** Designed for binary group comparisons. Definitions of "city center" and changes in group categorization may influence results	*Centralization*	*Race*	[79]
**Correlation Index** *(Correlation Ratio)*	The correlation index measures exposure between minority and majority group members and serves as an adjustment to the “Exposure Index” that accounts for its inherent asymmetry arising from the 2 comparison groups not having the same proportion of group members. The correlation index ranges from 0 to 1, with higher values indicating greater segregation and lower values indicating lesser segregation; a value of 0 indicates complete integration [77]	${\eta }^{2}= \frac{(I-P)}{(1-P)}$	I = The “Isolation Index” P = Proportion of the total minority population to the total population within an areal unit or study area	**STRENGTHS:** Accounts for asymmetry between groups and allows for comparisons across different contexts over time **LIMITATIONS:** Sensitive to group definitions and limited to binary groupings. Does not capture spatial distribution	*Exposure*	*Race*	[65]
**Local Spatial Segregation Index**	The Local Spatial Segregation Index (LSSI) measures the degree to which members of 2 groups are exposed to one another, providing a spatial measure of local segregation. LSSI is calculated for each area to capture the spatial segregation of minority group members from majority group members in a given area [65]. The index ranges from 0 to 1, corresponding to complete integration and complete segregation, respectively. In the case of 2 + comparison groups, LSSI can be easily modified to estimate an overall local value for the population [16]	${S}_{i}=1-\frac{({a}_{i}\sum \nolimits_{j}{c}_{ij}{b}_{j})+({b}_{i}\sum \nolimits_{j}{c}_{ij}{a}_{j})}{({a}_{i}\sum \nolimits_{j}{b}_{j})+({b}_{i}\sum \nolimits_{j}{a}_{j})}$	a = Population count group A in areal unit i b = Population count group B in areal unit i c = The value of cell ij in a spatial weights’ matrix, which equals 1 if areal units i and j share a border and zero otherwise (i.e., adjacency matrix)	**STRENGTHS:** Adjacency matrix accounts for shared borders between areal units which captures the spatial context of neighborhoods. Can handle multi-group comparisons **LIMITATIONS:** Sensitive to geographic scale and assumes homogeneous areas, which may not accurately represent the complexity of urban structures	*Exposure*	*Race*	[65]

The 5 composite indices identified by our review, cannot be classified according to Massey and Denton’s Dimensions of Residential Segregation [18], as they are based on several underlying factors, and encompass subjectively labeled domains, such as socioeconomic status. Moreover, the basis of comparison differs between SSP measures and composite indices; SSP measures compare polarization among privileged *and* deprived social or economic groups (e.g., both tails of a distribution), while composite indices compare social or economic position relative to privilege *or* deprivation (e.g., only one tail of a distribution). Considering these differences, SSP measures were examined separately from composite indices, with findings pertaining to the former displayed in Table 3, and the latter in the Supplementary Materials.

### SSP Measures

Our search yielded 18 distinct SSP measures (Table 3) across 7 domains: race, income, race/income, education, language, nativity, and home ownership. A majority of SSP measures focused on race, followed by income, and combined income/race. The domains of education, language, nativity, and home ownership were less frequently utilized.

The most commonly employed SSP measure was the Index of Concentration at the Extremes (ICE), which was used in 37% (*n* = 43) of included articles and applied to all of the above domains. The second most commonly employed SSP measure was the Index of Dissimilarity, followed by the Isolation Index, both of which were respectively featured in 18% (*n* = 21) and 17% (*n* = 20) of the evidence. Additional SSP measures include relative ratios (*n* = 9), the Local Getis Ord G* Statistic (*n* = 6), and the Gini Coefficient (*n* = 4). SSP measures such as the Location Quotient (*n* = 3), Redlining Index (*n* = 2), Entropy Index (*n* = 2), Delta Index (*n* = 2), and the Spatial Proximity Index (*n* = 2) were employed by only a handful of studies. Other SSP measures were less commonly employed, including the Exposure/Interaction Index (*n* = 1), Kernel Density Estimation (*n* = 1), the Atkinson Index (*n* = 1), the Krivo Local Isolation Index (*n* = 1), the Absolute Centralization Index (*n* = 1), the Correlation Index (*n* = 1), and the Local Spatial Segregation Index (*n* = 1).

#### SSP Measures of Concentration

The *Index of Concentration at the Extremes* (ICE) was the most frequently employed SSP measure in the body of evidence identified by our review, used in 43 studies. ICE was developed by Massey et al. [19] in 2001 to provide a single summary measure of economic polarization. ICE simultaneously captures SSP in both deprived and privileged social groups, and ranges from − 1 to 1, where negative values indicate greater deprivation, and positive values indicate greater privilege. More recently, Krieger et al. [26] extended ICE to capture both racial and economic polarization. Since then, ICE has emerged as a leading SSP measure in public health research [120]. Included studies have associated ICE with health outcomes including infant mortality [15, 27–32, 56], cancer [33–39], cardiovascular disease [8, 40, 55], injuries [41, 42, 57], premature mortality [3, 32, 43], and COVID-19 outcomes [58].

The *Location Quotient* measures the relative concentration of minority groups by comparing the proportion of minority group members in a smaller geographic unit (e.g., neighborhood) to the proportion of minority group members in a larger geographic unit (e.g., city). The Location Quotient can take on all non-negative real numbers, with higher values indicating a greater proportion of minority group members in the neighborhood compared to the entire city, and vice-versa. The Location Quotient was used to study breast cancer [109, 110], and colorectal cancer [111].

The *Delta Index* is another relative measure of concentration. The Delta Index ranges from 0 to 1 and represents the proportion of minority group members in a given geographic unit, that would have to move in order to achieve a uniform density across units. Note that this is similar to the evenness dimension, however, given the requisite of information on the land area occupied by each unit, and its explicit focus on density, the Delta Index captures spatial concentration instead of mere evenness. The Delta Index was used in 2 studies on sexually transmitted infections (STIs) during pregnancy [78] and self-rated health [79].

Additional SSP measures of concentration included *Relative Ratios*; a ratio comparing the frequency, probability, or odds of an event between a deprived (i.e., comparison) group and a privileged (i.e., referent) group. Relative Ratios include measures of association common to public health research like the risk ratio, rate ratio, and odds ratio. Relative Ratios range from − ∞ to ∞, and interpretation depends on the choice of comparison and referent group; however, Relative Ratios equal to 1 represent no difference between groups. Relative Ratios were used in 11 studies with various outcomes including non-communicable diseases [92, 93], general physical health [94, 95], and quality of life [44, 96]. The *Redlining Index* is similar to Relative Ratios; however, it measures the odds of mortgage loan denial and follows the interpretation of a pooled odds ratio. The Redlining Index was used in 2 studies on breast cancer survival [112] and preterm birth [64].

#### SSP Measures of Evenness

The *Index of Dissimilarity* was the second most frequently employed SSP measure, used in 22 studies. The Index of Dissimilarity is a popular measure of SSP, especially within the domain of racial residential segregation; it ranges from 0 to 1 and represents the proportion of a social group that would need to move across spatial units to achieve a uniform distribution. A Dissimilarity Index with a value of 0 indicates a uniform distribution (i.e., complete integration), and a value of 1 indicates complete segregation [65]. Since its development in 1955 [62], the Index of Dissimilarity has been clearly defined [121], and used in research on cancer [66, 67, 80], cardiovascular disease [68, 69, 81], STIs [70, 78], and obesity [65, 71].

The *Gini Coefficient* is a well-established measure of relative income inequality [122]. The Gini Coefficient indicates how the Lorenz curve, a cumulative frequency distribution, for a specific variable (e.g., income, race) deviates from its uniform distribution [123]. A coefficient of 0 represents perfect equality (i.e., all income is equally shared), and a coefficient of 1 represents perfect inequality (i.e., all income is earned by a single individual) [122]. The Gini Coefficient was used in 4 studies on outcomes including cancer [99], metabolic syndrome [45], and asthma [72], plus STIs [78]. The Gini Coefficient typically provides a summary statistic for a single variable but can be extended to accommodate two variables [98].

The Atkinson Index and the Entropy Index were less frequently used SSP measures of evenness. The *Atkinson Index* is similar to the above measures of evenness, as it was designed to evaluate SSP in terms of relative income inequality. Index values closer to 0 indicate an even income distribution (i.e., integration) and index values closer to 1 indicate an uneven income distribution (i.e., segregation). The Atkinson Index was recently used in 1 study to examine disparities in colorectal cancer [80]. The *Entropy Index* describes how the racial/ethnic diversity of spatial units within a city differs relative to the diversity (i.e., entropy) of the entire city. A value of 0 represents that all units have the same racial composition as the city, and a value of 1 represents that all units are composed of only 1 group. The Entropy Index was used in studies of self-rated health [79] and body mass index [65]. Both of the above measures include a sensitivity parameter that allows for differential weighting at different points along the distribution.

#### SSP Measures of Exposure

The *Isolation Index* was designed to measure the degree to which members of a minority group are exposed to other members of the minority group, based on the probability that minority group members share a geographic unit. An index of 0 indicates that a minority group member does not share a unit with another member of the same group, and an index of 1 indicates that the minority group member shares a unit with another member of the same minority group. The Isolation Index was used in 11 studies on cancer [82–84], cardiovascular disease [69, 81, 85, 86], mortality [64, 87], and COVID-19 [88].

The *Exposure/Interaction Index* is another measure of exposure. However, unlike the Isolation Index, the Exposure/Interaction Index describes the probability that a member of the minority group shares a geographic unit with a member of the majority group. This index ranges from 0 to 1, with lower values indicating greater segregation among groups and higher values indicating less segregation between groups. This measure was used in only 1 study which examined the association between the Exposure/Interaction Index and pre-term birth among pregnant women in Philadelphia, PA [64].

The *Krivo Local Isolation Index* encompasses both of the previously described SSP measures for the exposure dimension. The Krivo Local Isolation Index measures the probability of exposure between individuals belonging to 2 social groups compared with what would be expected for the entire city [100]. This index is not bounded between 0 and 1 and can include negative numbers; greater values of the Krivo Local Isolation Index indicate greater separation (i.e., less exposure/interaction) between the 2 social groups and vice-versa. The Krivo Local Isolation Index was used in 1 study which examined its association with low birth weight among singleton births [101].

The Correlation Index and the Local Spatial Segregation Index were less commonly employed; however, both offer information that is otherwise not captured using the above SSP measures for exposure. Similar to the Exposure/Interaction Index, the *Correlation Index* measures the relative exposure between minority and majority group members but provides an adjustment for the asymmetry inherent to the Exposure/Interaction Index that arises from relative differences in the size(s) of the groups being compared. The Correlation Index ranges from 0 to 1 with greater values indicating a greater probability of exposure between group members. The *Local Spatial Segregation Index* provides a snapshot of local segregation and can facilitate the comparison of more than 2 social groups. An index of 0 or 1 corresponds to the probability that members of the minority group are not exposed to/interacting with members of the majority group. Both indices were used in 1 study on food environments, racial segregation, and body mass index [65].

#### SSP Measures of Clustering

The Local Getis-Ord G* Statistic is a hot spot analysis method used to determine how the racial composition of a geographic unit (e.g., census tract) differs from that of neighboring units (e.g., adjacent census tracts) as compared to the mean racial composition for a larger geographic unit (e.g., city) [102]. The resulting z-scores and *p*-values guide interpretation with larger z-scores suggesting greater spatial clustering of higher values, and smaller z-scores suggesting greater spatial clustering of lower values. Statistical significance indicates that a unit with higher values is more likely to be adjacent to other units with similarly higher values than would be expected by chance. The Local Getis-Ord G* Statistic was employed in 6 studies on non-communicable diseases [103–106] and general physical health [107, 108].

The *Spatial Proximity Index* is an index designed to measure the spatial clustering of social groups [17]. The index represents the average intra-proximity between a minority group and a majority group, weighted by the proportion of social group members in the population. Spatial Proximity Index values greater than 1 indicate that minority group members reside closer to other minority group members than majority group members (i.e., greater clustering), and values less than 1 indicate that members of both the minority and majority group reside closer to each other, instead of residing near members of the same group. The Spatial Proximity Index was used in 2 studies on self-rated health [73, 79].

*Kernel Density Estimation* (KDE) is a method for identifying and mapping hot spots (i.e., clusters) that can be utilized to capture SSP. KDE identifies clustering by continuously applying a probability density function to spatial data and summing results to give a single KDE estimate which can then be used to determine the percent of group members at each population-weighted centroid, effectively providing a measure of clustering for a specified social group [117]. KDE was used in only 1 study on birth outcomes [118].

#### SSP Measures of Centralization

The *Absolute Centralization Index* was designed to measure how minority group members are distributed around the center of a given city. The Absolute Centralization Index ranges from − 1 to 1, with positive values suggesting greater centralization among minority group members, and negative values suggesting lesser centralization (e.g., living further from the city center) among minority group members. The Absolute Centralization Index can be extended to the *Relative Centralization Index* [18], which represents the relative proportion of minority group members that would have to move in order to achieve equivalent centralization with the majority group. The Absolute Centralization Index was used in 1 study on self-rated health [79].

## Discussion

In this scoping review, we reviewed evidence on the measurement of SSP and characterized the use of SSP measures in recent public health literature. Results from this review highlight 4 primary findings. First, we found that defining SSP in the context of privilege, deprivation, dissimilarity, and segregation returns a variety of distinct measures, each with its own interpretation. Second, we found a growing body of evidence that spanned various time periods, geographic settings, and health outcomes. Third, we demonstrated that a majority of SSP measures included in this review can be classified according to Massey and Denton’s Dimensions of Residential Segregation, which may ease the interpretability of this literature [18]. Fourth, we found articles with simultaneous attention to multiple SSP measures, as well as those focused on a single SSP measure. Last, we discuss considerations for the application of SSP measures in future public health research, highlighting the strengths, limitations, and contributions of our review.

Our search identified 23 measures overall, 18 of which were distinct measures of SSP, which we classified according to Massey and Denton’s dimensions, and 5 of which were composite indices, which are described in the Supplementary Materials. While SSP measures and composite indices are related, these measures differ on the basis of comparison—SSP measures capture both deprivation *and* privilege, while composite indices capture either deprivation *or* privilege, not both. This differentiation is a key element in developing a definition of SSP, which is a critical first step for investigations aiming to examine the impact of SSP on population health outcomes. Here, we propose such a definition of SSP, which requires capturing the relative distribution of the population on *both ends* of a polarized variable.

The majority of included studies were recent and set in the USA, with 44% published between 2020 and 2022 and 88% set in the USA. The most common study designs were cohort studies and cross-sectional studies. Studies included a variety of health outcomes, notably non-communicable diseases, cause-specific and all-cause mortality, general physical health, and maternal and perinatal health. There is a dearth of evidence on SSP and communicable diseases, which is surprising given the emergence of SARS-CoV-2 in early 2020 and the subsequent widening of health disparities across the US [124, 125]. Of the included evidence, only a handful of studies examined COVID-19 outcomes [58, 88, 126], including COVID-19 cases [58, 88], COVID-19 test positivity [58], and COVID-19 mortality [58, 126]. Considering how the COVID-19 pandemic impacted health and health equity [127], investigations featuring SSP may be instrumental in identifying and addressing the drivers of disparities in COVID-19 outcomes in various geographic units.

Classifying SSP measures according to Massey and Denton’s Dimensions of Residential Segregation revealed that concentration and evenness were the most frequently targeted dimensions, followed by exposure, clustering, and centralization. These dimensions were originally posited in Massey and Denton’s foundational 1988 manuscript [18], and the identifiability of each dimension was empirically re-confirmed by Massey, White, and Phua in 1996 [128], both of which were most recently discussed by Massey in 2012 [129]. Despite the ability to classify SSP measures using Massey and Denton’s Dimensions of Residential Segregation, we acknowledge that SSP is multidimensional in nature. Therefore, in agreement with Massey et al. [128, 129], we recommend that discussions surrounding SSP measurement move beyond those of selecting the “best” or “correct” SSP measure, and instead focus on a multidimensional approach based on several SSP measures.

In terms of the number of SSP measures employed by each study, we found that most studies included a single SSP measure (54.7%), several studies included 2 to 3 SSP measures (36.8%), and only 8.5% of studies included greater than 3 SSP measures. Of the studies that used more than 1 SSP measure, some examined the impact of using several distinct SSP measures, while others employed the same SSP measure across different domains.

### Guidance for Researchers

#### Selecting an SSP Measure

Selecting the appropriate SSP measure for a health study must be informed by the research question. As a multidimensional construct, researchers should identify which relevant dimensions (e.g., Massey and Denton’s) and domains (e.g., race, income) of SSP are of interest, to refine measures. Spatial scale matters; for small scales (e.g., census block), measures comparing unit demographics with broader segregation patterns (e.g., Location Quotient) may be preferable to within-unit measures (e.g., ICE). Researchers should also assess whether spatial autocorrelation is relevant, opting for measures leveraging it if needed (e.g., Krivo Local Isolation Index). Finally, researchers must ensure measures are interpretable for their intended audience.

#### SSP and Health Research Agenda

First, to move the SSP and health research agenda forward, we argue that a common definition of SSP is needed. Absent this, researchers should explicitly define the SSP motivating their work, using their own or our team’s conceptualization. Second, we identified mostly US-based studies, highlighting the need for non-US research to assess the applicability of these measures globally. Third, while we use Massey and Denton’s classifications (Table 1) to organize research, a health-focused taxonomy of SSP measures could guide future public health researchers in selecting appropriate measures. Fourth, SSP’s multidimensionality spans various domains and dimensions, supporting the use of multiple measures or one measure across domains [130]. Finally, future research should compare results across measures or domains and evaluate how measure selection affects findings [131].

### Strengths/Limitations

This scoping review has several limitations and strengths. Regarding limitations, the lack of an established SSP definition made the creation of an effective search strategy difficult. Although we reached a consensus on a definition for SSP while the review was in process, elaboration of a definition in advance may have informed additional terms to include in the search strategy. For example, the inclusion of an independent term for “polarization” may have been useful in identifying studies that employed the coefficient of polarization [132], an existing SSP measure not captured by our review. Additionally, though we did not restrict our search to the USA, our search strategy used US-based terms (tract, county, etc.), using more inclusive geographic terminology could have broadened our search strategy to capture more non-US studies. Regarding the strengths of this study, our scoping review provided a novel characterization of SSP measures and their application(s) in recent public health literature. We reviewed each SSP measure, compared measures in terms of their strengths and limitations, and provided tabulated results; all of which can help researchers navigate options for measuring SSP, and guide the selection of SSP measures for use in public health research.

## Conclusion

We conducted this scoping review to guide in the selection and application of SSP measures in public health research. We identified several unique SSP measures, their respective methods, and domains, and summarized their use in recent public health literature since 2007, filling a critical gap in the literature. Our findings draw attention to the benefits and pitfalls of each SSP measure and explore methodological options for measuring SSP in public health research. We also provide what we understand to be the first, provisional definition of SSP in the context of public health and highlight the importance of such a definition. Finally, aside from the resources offered in this review, the author team has developed the Spatial Social Polarization Database [133], an online application and interactive mapping tool, that can be used to examine select SSP measures, like ICE, at various geographies (https://drexel-uhc.shinyapps.io/SSP_Maps/), with a public repository [134]. We encourage researchers to leverage our findings and resources to better understand the role of SSP measurement in public health research, especially in the modern presence of both new and re-emerging health disparities.

## Supplementary Information

Below is the link to the electronic supplementary material.

Supplementary file1 (DOCX 69 KB)

## Data Availability

The data that support the findings of this study are available from the corresponding author, EMM, upon reasonable request.

## References

[1] Yang K, Qi H. Research on health disparities related to the COVID-19 pandemic: a bibliometric analysis. *Int J Environ Res Public Health*. 2022;19(3):1220. 35162243 10.3390/ijerph19031220 PMC8835299

[2] Diez Roux AV, Mair C. Neighborhoods and health. *Ann N Y Acad Sci.* 2010;1186:125–45. 10.1111/j.1749-6632.2009.05333.x. 10.1111/j.1749-6632.2009.05333.x 20201871

[3] Krieger N, Kim R, Feldman J, Waterman PD. Using the index of concentration at the extremes at multiple geographical levels to monitor health inequities in an era of growing spatial social polarization: massachusetts, USA (2010–14). *Int J Epidemiol*. 2018;47(3):788–819. 10.1093/ije/dyy004. 29522187 10.1093/ije/dyy004

[4] Mijs JJ, Roe EL. Is America coming apart? Socioeconomic segregation in neighborhoods, schools, workplaces, and social networks, 1970–2020. *Sociol Compass*. 2021;15(6): e12884.

[5] Wright R, Ellis M, Holloway SR, Wong S. Patterns of racial diversity and segregation in the United States: 1990–2010. *Prof Geogr*. 2014;66(2):173–82. 25083001 10.1080/00330124.2012.735924 PMC4114976

[6] Rothstein R. *The Color of Law: A Forgotten History of How Our Government Segregated America*. New York: Liveright Publishing Corporation; 2017.

[7] Trounstine J. *Segregation by design: local politics and inequality in American cities*. Cambridge: Cambridge University Press; 2019.

[8] Feldman JM, Waterman PD, Coull BA, Krieger N. Spatial social polarisation: using the index of concentration at the extremes jointly for income and race/ethnicity to analyse risk of hypertension. *J Epidemiol Community Health*. 2015;69(12):1199–207. 10.1136/jech-2015-205728. 26136082 10.1136/jech-2015-205728 PMC4878399

[9] van Ham M, Tammaru T, Ubareviéciençe R, Janssen H. *Urban socio-economic segregation and income inequality: a global perspective*. Cham: Springer; 2021.

[10] Sabatini F. *The social spatial segregation in the cities of Latin America*. United States of America: Inter-American Development Bank; [cited 2023 Jan 1]. Available from: https://coilink.org/20.500.12592/pg78wz. Accessed 01 Jan 2023

[11] Williams DR, Collins C. Racial residential segregation: a fundamental cause of racial disparities in health. *Public Health Rep*. 2001;116(5):404–16. 10.1093/phr/116.5.404. 12042604 10.1093/phr/116.5.404 PMC1497358

[12] Reeder L. Social epidemiology: an appraisal. *Patients, physicians and illness*. 1972;17(1):97–101.

[13] Massey D, Blaskeslee L. *An ecological perspective on assimilation and stratification*. Washington DC: American Sociological Association; 1983.

[14] Massey DS. Ethnic residential segregation: a theoretical synthesis and empirical review. *Sociol Soc Res*. 1985;69:315–50.

[15] Huynh M, Spasojevic J, Li W, et al. Spatial social polarization and birth outcomes: preterm birth and infant mortality - New York City, 2010–14. *Scandinavian journal of public health*. 2018;46(1):157–66. 10.1177/1403494817701566. 28385056 10.1177/1403494817701566

[16] Wong DW. Modeling local segregation: a spatial interaction approach. *Geogr Environ Model*. 2002;6(1):81–97.

[17] White MJ. The measurement of spatial segregation. *Am J Sociol*. 1983;88(5):1008–18.

[18] Massey DS, Denton NA. The dimensions of residential segregation. *Soc Forces*. 1988;67(2):281–315.

[19] Booth A, Crouter AC. *Does it take a village?: Community effects on children, adolescents, and families*. East Sussex: Psychology Press; 2001.

[20] Bailey ZD, Krieger N, Agénor M, Graves J, Linos N, Bassett MT. Structural racism and health inequities in the USA: evidence and interventions. *The Lancet*. 2017;389(10077):1453–63. 10.1016/S0140-6736(17)30569-X 28402827

[21] Innovation VH. Covidence systematic review software. Secondary Covidence systematic review software 2023. www.covidence.org. Accessed 01 Jan 2023

[22] Henson RM, Ortigoza A, Martinez-Folgar K, et al. Evaluating the health effects of place-based slum upgrading physical environment interventions: a systematic review (2012–2018). *Soc Sci Med*. 2020;261: 113102. 10.1016/j.socscimed.2020.113102[publishedOnlineFirst:20200615]. 32739786 10.1016/j.socscimed.2020.113102 PMC7611465

[23] Page MJ, McKenzie JE, Bossuyt PM, et al. The PRISMA 2020 statement: an updated guideline for reporting systematic reviews. *Int J Surg*. 2021;88: 105906. 33789826 10.1016/j.ijsu.2021.105906

[24] Tricco AC, Lillie E, Zarin W, et al. PRISMA extension for scoping reviews (PRISMA-ScR): checklist and explanation. *Ann Intern Med*. 2018;169(7):467–73. 30178033 10.7326/M18-0850

[25] Peters MDJ, Godfrey CM, Khalil H, McInerney P, Parker D, Soares CB. Guidance for conducting systematic scoping reviews. *Int J Evidence-Based Healthcare*. 2015;13(3):141. 10.1097/XEB.0000000000000050 26134548

[26] Krieger N, Waterman PD, Gryparis A, Coull BA. Black carbon exposure, socioeconomic and racial/ethnic spatial polarization, and the index of concentration at the extremes (ICE). *Health Place*. 2015;34:215–28. 10.1016/j.healthplace.2015.05.008. 26093080 10.1016/j.healthplace.2015.05.008 PMC4681506

[27] Chambers BD, Baer RJ, McLemore MR, Jelliffe-Pawlowski LL. Using index of concentration at the extremes as indicators of structural racism to evaluate the association with preterm birth and infant mortality-California, 2011–2012. *J Urban Health : Bulletin of the New York Academy of Medicine*. 2019;96(2):159–70. 10.1007/s11524-018-0272-4. 10.1007/s11524-018-0272-4 PMC6458187 29869317

[28] Eick SM, Cushing L, Goin DE, et al. Neighborhood conditions and birth outcomes: understanding the role of perceived and extrinsic measures of neighborhood quality. *Environ Epidemiol*. 2022;6(5): e224. 10.1097/ee9.0000000000000224. 36249266 10.1097/EE9.0000000000000224 PMC9555921

[29] Fong KC, Yitshak-Sade M, Lane KJ, et al. Racial disparities in associations between neighborhood demographic polarization and birth weight. *Int J Environ Res Public Health*. 2020;17(9):3076. 10.3390/ijerph17093076. 32354151 10.3390/ijerph17093076 PMC7246784

[30] Janevic T, Zeitlin J, Egorova NN, et al. Racial and economic neighborhood segregation, site of delivery, and morbidity and mortality in neonates born very preterm. *J Pediatr*. 2021;235:116–23. 10.1016/j.jpeds.2021.03.049. 33794221 10.1016/j.jpeds.2021.03.049 PMC9582630

[31] Krieger N, Waterman PD, Batra N, Murphy JS, Dooley DP, Shah SN. Measures of local segregation for monitoring health inequities by local health departments. *Am J Public Health*. 2017;107(6):903–6. 10.2105/ajph.2017.303713. 28426303 10.2105/AJPH.2017.303713 PMC5425857

[32] Krieger N, Waterman PD, Spasojevic J, Li W, Maduro G, Van Wye G. Public health monitoring of privilege and deprivation with the index of concentration at the extremes. *Am J Public Health*. 2016;106(2):256–63. 10.2105/ajph.2015.302955. 26691119 10.2105/AJPH.2015.302955 PMC4815605

[33] Connor AE, Kaur M, Dibble KE, Visvanathan K, Dean LT, Hayes JH. Racialized economic segregation and breast cancer mortality among women in Maryland. *Cancer Epidemiol, Biomarkers & Prevention*. 2022;31(2):413–21. 10.1158/1055-9965.Epi-21-0923. 10.1158/1055-9965.EPI-21-0923 PMC8825681 34862211

[34] Goel N, Westrick AC, Bailey ZD, et al. Structural racism and breast cancer-specific survival: impact of economic and racial residential segregation. *Ann Surg*. 2022;275(4):776–83. 10.1097/sla.0000000000005375. 35081560 10.1097/SLA.0000000000005375 PMC9102835

[35] Krieger N, Feldman JM, Kim R, Waterman PD. Cancer incidence and multilevel measures of residential economic and racial segregation for cancer registries. *JNCI Cancer Spectr*. 2018;2(1):pky09. 10.1093/jncics/pky009. 10.1093/jncics/pky009 PMC6649696 31360840

[36] Krieger N, Wright E, Chen JT, Waterman PD, Huntley ER, Arcaya M. Cancer stage at diagnosis, historical redlining, and current neighborhood characteristics: breast, cervical, lung, and colorectal cancers, Massachusetts, 2001–2015. *Am J Epidemiol*. 2020;189(10):1065–75. 10.1093/aje/kwaa045. 32219369 10.1093/aje/kwaa045 PMC7666416

[37] Siegel SD, Brooks MM, Lynch SM, Sims-Mourtada J, Schug ZT, Curriero FC. Racial disparities in triple negative breast cancer: toward a causal architecture approach. *Breast cancer research : bCR*. 2022;24(1):37. 10.1186/s13058-022-01533-z. 35650633 10.1186/s13058-022-01533-z PMC9158353

[38] Westrick AC, Bailey ZD, Schlumbrecht M, et al. Residential segregation and overall survival of women with epithelial ovarian cancer. *Cancer*. 2020;126(16):3698–707. 10.1002/cncr.32989. 32484923 10.1002/cncr.32989

[39] Wiese D, Stroup AM, Crosbie A, Lynch SM, Henry KA. The impact of neighborhood economic and racial inequalities on the spatial variation of breast cancer survival in New Jersey. *Cancer Epidemiol, Biomarkers & Prevention*. 2019;28(12):1958–67. 10.1158/1055-9965.Epi-19-0416. 10.1158/1055-9965.EPI-19-0416 31649136

[40] Feldman JM, Conderino S, Islam NS, Thorpe LE. Subgroup variation and neighborhood social gradients-an analysis of hypertension and diabetes among Asian patients (New York City, 2014–2017). *J Racial Ethn Health Disparities*. 2021;8(1):256–63. 10.1007/s40615-020-00779-7. 32488823 10.1007/s40615-020-00779-7 PMC7708414

[41] Schleimer JP, Buggs SA, McCort CD, et al. Neighborhood racial and economic segregation and disparities in violence during the COVID-19 pandemic. *Am J Public Health*. 2022;112(1):144–53. 10.2105/ajph.2021.306540. 34882429 10.2105/AJPH.2021.306540 PMC8713621

[42] Krieger N, Feldman JM, Waterman PD, Chen JT, Coull BA, Hemenway D. Local residential segregation matters: stronger association of census tract compared to conventional city-level measures with fatal and non-fatal assaults (total and firearm related), using the index of concentration at the extremes (ICE) for racial, economic, and racialized economic segregation, Massachusetts (US), 1995–2010. *J Urban Health : Bull New York Acad Med*. 2017;94(2):244–58. 10.1007/s11524-016-0116-z. 10.1007/s11524-016-0116-z PMC5391325 28130678

[43] Lange-Maia BS, De Maio F, Avery EF, et al. Association of community-level inequities and premature mortality: chicago, 2011–2015. *J Epidemiol Community Health*. 2018;72(12):1099–103. 10.1136/jech-2018-210916. 30171083 10.1136/jech-2018-210916

[44] Casciano R, Massey DS. Neighborhoods, employment, and welfare use: assessing the influence of neighborhood socioeconomic composition. *Soc Sci Res*. 2008;37(2):544–58. 10.1016/j.ssresearch.2007.08.008. 19069058 10.1016/j.ssresearch.2007.08.008 PMC3710739

[45] Pichardo CM, Pichardo MS, Gallo LC, et al. Association of neighborhood segregation with 6-year incidence of metabolic syndrome in the Hispanic community health study/study of Latinos. *Ann Epidemiol*. 2022;78:1–8. 10.1016/j.annepidem.2022.11.003. 10.1016/j.annepidem.2022.11.003 PMC10127516 36473628

[46] Abbott EE, Buckler DG, Hsu JY, et al. Survival after out-of-hospital cardiac arrest: the role of racial residential segregation. *J Urban Health : Bull New York Acad Med*. 2022;99(6):998–1011. 10.1007/s11524-022-00691-x. 10.1007/s11524-022-00691-x PMC9727016 36216971

[47] Acker J, Mujahid M, Aghaee S, et al. Neighborhood racial and economic privilege and timing of pubertal onset in girls. *J Adolesc Health*. 2022. 10.1016/j.jadohealth.2022.10.013. 10.1016/j.jadohealth.2022.10.013 PMC10505041 36528517

[48] Carpiano RM, Lloyd JE, Hertzman C. Concentrated affluence, concentrated disadvantage, and children's readiness for school: a population-based, multi-level investigation. *Soc Sci Med*. 2009;69(3):420–32. 10.1016/j.socscimed.2009.05.028. 10.1016/j.socscimed.2009.05.028 19540643

[49] Elser H, Rowland ST, Tartof SY, et al. Ambient temperature and risk of urinary tract infection in California: A time-stratified case-crossover study using electronic health records. *Environ Int*. 2022;165:107303. 10.1016/j.envint.2022.107303. 35635960 10.1016/j.envint.2022.107303 PMC9233468

[50] Feldman JM, Gruskin S, Coull BA, Krieger N. Police-related deaths and neighborhood economic and racial/ethnic polarization, United States, 2015-2016. *Am J Public Health*. 2019;109(3):458–64. 10.2105/ajph.2018.304851. 30676802 10.2105/AJPH.2018.304851 PMC6366529

[51] Hruska B, Pacella-LaBarbara ML, Castro IE, George RL, Delahanty DL. Incorporating community-level risk factors into traumatic stress research: adopting a public health lens. *J Anxiety Disord*. 2022;86:102529. 10.1016/j.janxdis.2022.102529. 35074683 10.1016/j.janxdis.2022.102529

[52] Shrimali BP, Pearl M, Karasek D, Reid C, Abrams B, Mujahid M. Neighborhood privilege, preterm delivery, and related racial/ethnic disparities: an intergenerational application of the index of concentration at the extremes. *Am J Epidemiol*. 2020;189(5):412–21. 10.1093/aje/kwz279. 31909419 10.1093/aje/kwz279

[53] Shumate C, Hoyt A, Liu C, Kleinert A, Canfield M. Understanding how the concentration of neighborhood advantage and disadvantage affects spina bifida risk among births to non-Hispanic white and Hispanic women, Texas, 1999-2014. *Birth Defects Res*. 2019;111(14):982–90. 10.1002/bdr2.1374. 30198630 10.1002/bdr2.1374

[54] Smith LB, O'Brien C, Kenney GM, et al. Racialized economic segregation and potentially preventable hospitalizations among Medicaid/CHIP-enrolled children. *Health Serv Res*. 2022. 10.1111/1475-6773.14120. 10.1111/1475-6773.14120 PMC10154153 36527452

[55] Yitshak-Sade M, Lane KJ, Fabian MP, et al. Race or racial segregation? Modification of the PM2.5 and cardiovascular mortality association. *PLOS ONE*. 2020;15(7):e0236479. 10.1371/journal.pone.0236479. 32716950 10.1371/journal.pone.0236479 PMC7384646

[56] Karvonen KL, McKenzie-Sampson S, Baer RJ, et al. Structural racism is associated with adverse postnatal outcomes among Black preterm infants. *Pediatr Res*. 2022;94:371. 10.1038/s41390-022-02445-6. 36577795 10.1038/s41390-022-02445-6 PMC9795138

[57] Jay J, Kondo MC, Lyons VH, Gause E, South EC. Neighborhood segregation, tree cover and firearm violence in 6 U.S. cities, 2015-2020. *Preventive Med*. 2022;165(1):107256. 10.1016/j.ypmed.2022.107256. 10.1016/j.ypmed.2022.107256 PMC10903784 36115422

[58] Chen JT, Krieger N. Revealing the unequal burden of COVID-19 by income, race/ethnicity, and household crowding: US county versus zip code analyses. *J Public Health Manag Pract*. 2021;27(Supplement 1):S43-56. 10.1097/phh.0000000000001263. 10.1097/PHH.0000000000001263 32956299

[59] Cushing L, Morello-Frosch R, Hubbard A. Extreme heat and its association with social disparities in the risk of spontaneous preterm birth. *Paediatr Perinat Epidemiol*. 2022;36(1):13–22. 10.1111/ppe.12834. 34951022 10.1111/ppe.12834

[60] Dyer L, Chambers BD, Crear-Perry J, Theall KP, Wallace M. The index of concentration at the extremes (ICE) and pregnancy-associated mortality in Louisiana, 2016-2017. *Matern Child Health J*. 2022;26(4):814–22. 10.1007/s10995-021-03189-1. 34148221 10.1007/s10995-021-03189-1 PMC8684557

[61] Ward JB, Albrecht SS, Robinson WR, et al. Neighborhood language isolation and depressive symptoms among elderly U.S. Latinos. *Ann Epidemiol*. 2018;28(11):774–82. 10.1016/j.annepidem.2018.08.009. 30201290 10.1016/j.annepidem.2018.08.009 PMC6215715

[62] Duncan OD, Duncan B. A methodological analysis of segregation indexes. *Am Sociol Rev*. 1955;20(2):210–7.

[63] Kramer MR, Cooper HL, Drews-Botsch CD, Waller LA, Hogue CR. Do measures matter? Comparing surface-density-derived and census-tract-derived measures of racial residential segregation. *Int J Health Geogr*. 2010;9:1–15. 20540797 10.1186/1476-072X-9-29 PMC2898812

[64] Mendez DD, Hogan VK, Culhane JF. Institutional racism, neighborhood factors, stress, and preterm birth. *Ethn Health*. 2014;19(5):479–99. 10.1080/13557858.2013.846300. 24134165 10.1080/13557858.2013.846300 PMC11219028

[65] Goodman M, Lyons S, Dean LT, Arroyo C, Hipp JA. How segregation makes us fat: food behaviors and food environment as mediators of the relationship between residential segregation and individual body mass index. *Front Public Health*. 2018;6:92. 10.3389/fpubh.2018.00092. 29651414 10.3389/fpubh.2018.00092 PMC5884945

[66] Annesi CA, Poulson MR, Mak KS, et al. The impact of residential racial segregation on non-small cell lung cancer treatment and outcomes. *Ann Thorac Surg*. 2022;113(4):1291–8. 10.1016/j.athoracsur.2021.04.096. 34033745 10.1016/j.athoracsur.2021.04.096

[67] Blanco BA, Poulson M, Kenzik KM, McAneny DB, Tseng JF, Sachs TE. The impact of residential segregation on pancreatic cancer diagnosis, treatment, and mortality. *Ann Surg Oncol*. 2021;28(6):3147–55. 10.1245/s10434-020-09218-7. 33135144 10.1245/s10434-020-09218-7

[68] Gaglioti AH, Rivers D, Ringel JB, Judd S, Safford MM. Individual and neighborhood influences on the relationship between waist circumference and coronary heart disease in the reasons for geographic and racial differences in stroke study. *Prev Chronic Dis*. 2022;19:E20. 10.5888/pcd19.210195. 35446759 10.5888/pcd19.210195 PMC9044900

[69] Sarrazin MS, Campbell ME, Richardson KK, Rosenthal GE. Racial segregation and disparities in health care delivery: conceptual model and empirical assessment. *Health Serv Res*. 2009;44(4):1424–44. 10.1111/j.1475-6773.2009.00977.x. 19467026 10.1111/j.1475-6773.2009.00977.x PMC2739036

[70] Ford CL, Daniel M, Earp JA, Kaufman JS, Golin CE, Miller WC. Perceived everyday racism, residential segregation, and HIV testing among patients at a sexually transmitted disease clinic. *Am J Public Health*. 2009;99(Suppl 1):S137-43. 10.2105/ajph.2007.120865. 19218186 10.2105/AJPH.2007.120865 PMC2724930

[71] Do DP, Frank R. The diverging impacts of segregation on obesity risk by nativity and neighborhood poverty among Hispanic Americans. *J Racial Ethn Health Disparities*. 2020;7(6):1214–24. 10.1007/s40615-020-00746-2. 32291576 10.1007/s40615-020-00746-2

[72] Shankardass K, Jerrett M, Milam J, Richardson J, Berhane K, McConnell R. Social environment and asthma: associations with crime and no child left behind programmes. *J Epidemiol Community Health*. 2011;65(10):859–65. 10.1136/jech.2009.102806. 21071562 10.1136/jech.2009.102806 PMC4384703

[73] Do DP, Frank R, Iceland J. Black-white metropolitan segregation and self-rated health: investigating the role of neighborhood poverty. *Soc Sci Med*. 1982;2017(187):85–92. 10.1016/j.socscimed.2017.06.010. 10.1016/j.socscimed.2017.06.010 28667834

[74] Chandola T, Mikkilineni S, Chandran A, Bandyopadhyay SK, Zhang N, Bassanesi SL. Is socioeconomic segregation of the poor associated with higher premature mortality under the age of 60? A cross-sectional analysis of survey data in major Indian cities. *BMJ Open*. 2018;8(2):e018885. 10.1136/bmjopen-2017-018885. 29440157 10.1136/bmjopen-2017-018885 PMC5829777

[75] Bell W. A probability model for the measurement for ecological segregation. *Soc F*. 1953;32:357.

[76] Lieberson S, Carter DK. Temporal changes and urban differences in residential segregation: a reconsideration. *Am J Sociol*. 1982;88(2):296–310.

[77] US Census Bureau. Housing patterns. Appendix B: measures of residential segregation 2021.https://www.census.gov/topics/housing/housing-patterns/guidance/appendix-b.html. Accessed 14 Sept 2023

[78] Noah AJ, Yang TC, Wang WL. The Black-White disparity in sexually transmitted diseases during pregnancy: how do racial segregation and income inequality matter? *Sex Transm Dis*. 2018;45(5):301–6. 10.1097/olq.0000000000000820. 29485542 10.1097/OLQ.0000000000000820 PMC5895497

[79] Yang TC, Zhao Y, Song Q. Residential segregation and racial disparities in self-rated health: how do dimensions of residential segregation matter? *Soc Sci Res*. 2017;61:29–42. 10.1016/j.ssresearch.2016.06.011. 27886735 10.1016/j.ssresearch.2016.06.011 PMC5124442

[80] Leslie TF, Frankenfeld CL, Menon N. Disparities in colorectal cancer time-to-treatment and survival time associated with racial and economic residential segregation surrounding the diagnostic hospital, Georgia 2010–2015. *Cancer Epidemiol*. 2022;81: 102267. 10.1016/j.canep.2022.102267. 36166941 10.1016/j.canep.2022.102267

[81] Kershaw KN, Diez Roux AV, Burgard SA, Lisabeth LD, Mujahid MS, Schulz AJ. Metropolitan-level racial residential segregation and Black-White disparities in hypertension. *Am J Epidemiol*. 2011;174(5):537–45. 10.1093/aje/kwr116. 21697256 10.1093/aje/kwr116 PMC3202148

[82] Haas JS, Earle CC, Orav JE, et al. Racial segregation and disparities in breast cancer care and mortality. *Cancer*. 2008;113(8):2166–72. 10.1002/cncr.23828. 18798230 10.1002/cncr.23828 PMC2575036

[83] Haas JS, Earle CC, Orav JE, Brawarsky P, Neville BA, Williams DR. Racial segregation and disparities in cancer stage for seniors. *J Gen Intern Med*. 2008;23(5):699–705. 10.1007/s11606-008-0545-9. 18338215 10.1007/s11606-008-0545-9 PMC2324162

[84] Johnson AM, Johnson A, Hines RB, Mohammadi R. Neighborhood context and non-small cell lung cancer outcomes in Florida non-elderly patients by race/ethnicity. *Lung Cancer*. 2020;142:20–7. 10.1016/j.lungcan.2020.01.012. 32062478 10.1016/j.lungcan.2020.01.012

[85] Bravo MA, Batch BC, Miranda ML. Residential racial isolation and spatial patterning of hypertension in Durham. *North Carolina Preventing chronic disease*. 2019;16:E36. 10.5888/pcd16.180445. 30925142 10.5888/pcd16.180445 PMC6464129

[86] Greer S, Casper M, Kramer M, et al. Racial residential segregation and stroke mortality in Atlanta. *Ethn Dis*. 2011;21(4):437–43. 22428347

[87] Anthopolos R, Kaufman JS, Messer LC, Miranda ML. Racial residential segregation and preterm birth: built environment as a mediator. *Epidemiology*. 2014;25(3):397–405. 10.1097/ede.0000000000000079. 24681575 10.1097/EDE.0000000000000079

[88] Hu T, Yue H, Wang C, et al. Racial segregation, testing site access, and COVID-19 incidence rate in Massachusetts, USA. *Int J Environ Res Public Health*. 2020;17(24):9528. 10.3390/ijerph17249528. 33352650 10.3390/ijerph17249528 PMC7766428

[89] Do DP, Locklar LRB, Florsheim P. Triple jeopardy: the joint impact of racial segregation and neighborhood poverty on the mental health of black Americans. *Soc Psychiatry Psychiatr Epidemiol*. 2019;54(5):533–41. 10.1007/s00127-019-01654-5. 30671599 10.1007/s00127-019-01654-5

[90] Bravo MA, Anthopolos R, Kimbro RT, Miranda ML. Residential racial isolation and spatial patterning of type 2 diabetes mellitus in Durham, North Carolina. *Am J Epidemiol*. 2018;187(7):1467–76. 10.1093/aje/kwy026. 29762649 10.1093/aje/kwy026

[91] Woo H, Brigham EP, Allbright K, et al. Racial segregation and respiratory outcomes among urban black residents with and at risk of chronic obstructive pulmonary disease. *Am J Respir Crit Care Med*. 2021;204(5):536–45. 10.1164/rccm.202009-3721OC. 33971109 10.1164/rccm.202009-3721OC PMC8491265

[92] Cole H, Duncan DT, Ogedegbe G, Bennett S, Ravenell J. Neighborhood socioeconomic disadvantage; neighborhood racial composition; and hypertension stage, awareness, and treatment among hypertensive Black men in New York city: does nativity matter? *J Racial Ethn Health Disparities*. 2016. 10.1007/s40615-016-0289-x. 27659485 10.1007/s40615-016-0289-x PMC5362363

[93] Cozier YC, Yu J, Coogan PF, Bethea TN, Rosenberg L, Palmer JR. Racism, segregation, and risk of obesity in the Black women’s health study. *Am J Epidemiol*. 2014;179(7):875–83. 10.1093/aje/kwu004. 24585257 10.1093/aje/kwu004 PMC3969538

[94] Borrell LN, Kiefe CI, Diez-Roux AV, Williams DR, Gordon-Larsen P. Racial discrimination, racial/ethnic segregation, and health behaviors in the CARDIA study. *Ethn Health*. 2013;18(3):227–43. 10.1080/13557858.2012.713092. 22913715 10.1080/13557858.2012.713092 PMC3523091

[95] Gibbons J, Yang TC. Self-rated health and residential segregation: how does race/ethnicity matter? *J Urban Health : Bull New York Acad Med*. 2014;91(4):648–60. 10.1007/s11524-013-9863-2. 10.1007/s11524-013-9863-2 PMC4134452 24515933

[96] Ludwig J, Duncan GJ, Gennetian LA, et al. Neighborhood effects on the long-term well-being of low-income adults. *Science*. 2012;337(6101):1505–10. 10.1126/science.1224648. 22997331 10.1126/science.1224648 PMC3491569

[97] Ceriani L, Verme P. The origins of the Gini index: extracts from Variabilità e Mutabilità (1912) by Corrado Gini. *J Econ Inequal*. 2012;10:421–43.

[98] Brown MC. Using Gini-style indices to evaluate the spatial patterns of health practitioners: theoretical considerations and an application based on Alberta data. *Soc Sci Med*. 1994;38(9):1243–56. 8016689 10.1016/0277-9536(94)90189-9

[99] Plascak JJ, Llanos AA, Pennell ML, Weier RC, Paskett ED. Neighborhood factors associated with time to resolution following an abnormal breast or cervical cancer screening test. *Cancer Epidemiol, Biomarkers & Prevention*. 2014;23(12):2819–28. 10.1158/1055-9965.Epi-14-0348. 10.1158/1055-9965.EPI-14-0348 PMC4257881 25205516

[100] Krivo LJ, Byron RA, Calder CA, et al. Patterns of local segregation: do they matter for neighborhood crime? *Soc Sci Res*. 2015;54:303–18. 26463550 10.1016/j.ssresearch.2015.08.005

[101] Debbink MP, Bader MD. Racial residential segregation and low birth weight in Michigan’s metropolitan areas. *Am J Public Health*. 2011;101(9):1714–20. 10.2105/ajph.2011.300152. 21778487 10.2105/AJPH.2011.300152 PMC3154240

[102] Monzur T.* Local G statistics or Getis ord Gi* in analysing spatial pattern*. Kyoto: Asia Pacific University; 2015. p. 1–6. Available from: 10.13140/RG.2.1.1431.2402.

[103] Bancks MP, Kershaw K, Carson AP, Gordon-Larsen P, Schreiner PJ, Carnethon MR. Association of modifiable risk factors in young adulthood with racial disparity in incident type 2 diabetes during middle adulthood. *JAMA*. 2017;318(24):2457–65. 10.1001/jama.2017.19546. 29279935 10.1001/jama.2017.19546 PMC5820714

[104] Buehler JW, Castro JC, Cohen S, Zhao Y, Melly S, Moore K. Personal and neighborhood attributes associated with cervical and colorectal cancer screening in an urban African American population. *Prev Chronic Dis*. 2019;16:E118. 10.5888/pcd16.190030. 31469069 10.5888/pcd16.190030 PMC6716424

[105] Lê-Scherban F, Ballester L, Castro JC, et al. Identifying neighborhood characteristics associated with diabetes and hypertension control in an urban African-American population using geo-linked electronic health records. *Prev Med Rep*. 2019;15: 100953. 10.1016/j.pmedr.2019.100953. 31367515 10.1016/j.pmedr.2019.100953 PMC6656692

[106] Mayne SL, Hicken MT, Merkin SS, et al. Neighbourhood racial/ethnic residential segregation and cardiometabolic risk: the multiethnic study of atherosclerosis. *J Epidemiol Community Health*. 2019;73(1):26–33. 10.1136/jech-2018-211159. 30269056 10.1136/jech-2018-211159 PMC6398328

[107] Wang G, Schwartz GL, Kershaw KN, McGowan C, Kim MH, Hamad R. The association of residential racial segregation with health among U.S. children: a nationwide longitudinal study. *SSM Popul Health*. 2022;19:101250. 10.1016/j.ssmph.2022.101250. 36238814 10.1016/j.ssmph.2022.101250 PMC9550534

[108] Schwartz GL, Wang G, Kershaw KN, McGowan C, Kim MH, Hamad R. The long shadow of residential racial segregation: associations between childhood residential segregation trajectories and young adult health among Black US Americans. *Health Place*. 2022;77: 102904. 10.1016/j.healthplace.2022.102904. 36063651 10.1016/j.healthplace.2022.102904 PMC10166594

[109] Bonner SN, Clark C, Keating NL, Kouri EM, Freedman RA. Examining associations of racial residential segregation with patient knowledge of breast cancer and treatment receipt. *Clin Breast Cancer*. 2019;19(3):178-87.e3. 10.1016/j.clbc.2018.12.001. 10.1016/j.clbc.2018.12.001 PMC6556145 30685264

[110] Pruitt SL, Lee SJ, Tiro JA, Xuan L, Ruiz JM, Inrig S. Residential racial segregation and mortality among black, white, and Hispanic urban breast cancer patients in Texas, 1995 to 2009. *Cancer*. 2015;121(11):1845–55. 10.1002/cncr.29282. 25678448 10.1002/cncr.29282 PMC5308210

[111] Zhou Y, Bemanian A, Beyer KM. Housing discrimination, residential racial segregation, and colorectal cancer survival in southeastern Wisconsin. *Cancer Epidemiol, Biomarkers & Prevention*. 2017;26(4):561–8. 10.1158/1055-9965.Epi-16-0929. 10.1158/1055-9965.EPI-16-0929 28196847

[112] Beyer KM, Zhou Y, Matthews K, Bemanian A, Laud PW, Nattinger AB. New spatially continuous indices of redlining and racial bias in mortgage lending: links to survival after breast cancer diagnosis and implications for health disparities research. *Health Place*. 2016;40:34–43. 10.1016/j.healthplace.2016.04.014. 27173381 10.1016/j.healthplace.2016.04.014

[113] Theil H, Finizza AJ. *A note on the measurement of racial integration of schools by means of informational concepts*. Springer; 1971.

[114] Hoover EM. Interstate redistribution of population, 1850–1940. *J Econ Hist*. 1941;1(2):199–205.

[115] Duncan OD, Ray PC, Beverly D. *Statistical geography: problems in analyzing areal data*: Free Press; 1961.

[116] Peach C, Robinson V, Smith S. *Ethnic segregation in cities*: Taylor & Francis; 2023.

[117] Hu Y, Wang F, Guin C, Zhu H. A spatio-temporal kernel density estimation framework for predictive crime hotspot mapping and evaluation. *Appl Geogr*. 2018;99:89–97.

[118] Planey AM, Grady SC, Fetaw R, McLafferty SL. Spaces of segregation and health: complex associations for Black immigrant and US-born mothers in New York city. *J Urban Health : Bull New York Acad Med*. 2022;99(3):469–81. 10.1007/s11524-022-00634-6. 10.1007/s11524-022-00634-6 PMC9187803 35486284

[119] Atkinson AB. On the measurement of inequality. *J Econ Theory*. 1970;2(3):244–63.

[120] LarrabeeSonderlund A, Charifson M, Schoenthaler A, Carson T, Williams NJ. Racialized economic segregation and health outcomes: a systematic review of studies that use the index of concentration at the extremes for race, income, and their interaction. *PLoS ONE*. 2022;17(1): e0262962. 35089963 10.1371/journal.pone.0262962 PMC8797220

[121] Forest B. Measures of segregation and isolation. *Dartmouth College*. 2005;8(12):2016.

[122] De Maio FG. Income inequality measures. *J Epidemiol Community Health*. 2007;61(10):849. 17873219 10.1136/jech.2006.052969 PMC2652960

[123] Schneider MC, Castillo-Salgado C, Bacallao J, Loyola E, Mujica OJ, Vidaurre M, Roca A. Summary of indicators most used for the measurement of the health inequalities. *Epidemiol bull*. 2005;26(3):7–10. 16578907

[124] Bilal U, Mullachery P, Schnake-Mahl A, et al. Heterogeneity in the spatial Inequities in COVID-19 vaccination in across 16 large US cities. *Am J Epidemiol*. 2022. 10.1093/aje/kwac076[publishedOnlineFirst:20220422]. 35452081 10.1093/aje/kwac076 PMC9047229

[125] Diez Roux A, Kolker J, Barber S, Bilal U, Mullachery P, Schnake-Mahl A, McCulley E, Vaidya V, Ran L, Rollins H, Furukawa A, Koh C, Sharaf A, Dureja K, O’Sullivan C, Gibson A. *COVID-19 health inequities in cities dashboard*. Urban Health Collaborative: Drexel University; 2021.

[126] Khanijahani A, Tomassoni L. Socioeconomic and racial segregation and COVID-19: concentrated disadvantage and Black concentration in association with COVID-19 deaths in the USA. *J Racial Ethn Health Disparities*. 2022;9(1):367–75. 10.1007/s40615-021-00965-1. 33469872 10.1007/s40615-021-00965-1 PMC7815201

[127] Shadmi E, Chen Y, Dourado I, et al. Health equity and COVID-19: global perspectives. *Int J Equity Health*. 2020;19(1):1–16. 10.1186/s12939-020-01218-z PMC7316580 32586388

[128] Massey DS, White MJ, Phua V-C. The dimensions of segregation revisited. *Sociol Methods Res*. 1996;25(2):172–206.

[129] Massey DS. Reflections on the dimensions of segregation. *Soc Forces*. 2012;91(1):39–43. 24920864 10.1093/sf/sos118 PMC4049562

[130] Yang TC, Park K, Matthews SA. Racial/ethnic segregation and health disparities: future directions and opportunities. *Sociol Compass*. 2020;14(6): e12794. 32655686 10.1111/soc4.12794 PMC7351362

[131] Bemanian A, Beyer KM. Measures matter: the local exposure/isolation (LEx/Is) metrics and relationships between local-level segregation and breast cancer survival. *Cancer Epidemiol Biomarkers Prev*. 2017;26(4):516–24. 10.1158/1055-9965.EPI-16-0926 PMC5380473 28325737

[132] Walks A, Twigge-Molecey A. *Income inequality and polarization in Canada's cities: An examination and new form of measurement*. Toronto: University of Toronto; 2014.

[133] Freuh L, Jaros S, Abdel Magid HS, Lovasi GS. *Spatial social polarization database: an interactive mapping tool* [Internet]. Drexel University Urban Health Collaborative; 2024. Available from: https://drexel-uhc.shinyapps.io/SSP_Maps/. Accessed Oct 2024

[134] Jaros S. *Spatial social polarization database repository*. GitHub; 2024. Available from: https://github.com/samjaros-stanford/spatial_social_polarization_database. Accessed 10 Oct 2024

[135] Burris HH, Mullin AM, Dhudasia MB, et al. Neighborhood characteristics and racial disparities in severe acute respiratory syndrome coronavirus 2 (SARS-CoV-2) seropositivity in pregnancy. *Obstet Gynecol* 2022;139(6):1018–26. 10.1097/aog.0000000000004791 10.1097/AOG.0000000000004791 PMC9180815 35675599

[136] Leapman MS, Dinan M, Pasha S, et al. Mediators of racial disparity in the use of prostate magnetic resonance imaging among patients with prostate cancer. *JAMA Oncol* 2022;8(5):687–96. 10.1001/jamaoncol.2021.8116. 10.1001/jamaoncol.2021.8116 PMC8895315 35238879

[137] Linton SL, Cooper HLF, Chen YT, et al. Mortgage discrimination and racial/ethnic concentration are associated with same-race/ethnicity partnering among people who inject drugs in 19 US cities. *J Urban Health* 2020;97(1):88–104. 10.1007/s11524-019-00405-w. 10.1007/s11524-019-00405-w PMC7010885 31933055

[138] Wadhwani SI, Brokamp C, Rasnick E, Bucuvalas JC, Lai JC, Beck AF. Neighborhood socioeconomic deprivation, racial segregation, and organ donation across 5 states. *Am J Transplant* 2021;21(3):1206–14. 10.1111/ajt.16186. 10.1111/ajt.16186 PMC8191504 32654392

[139] Harvey VM, Enos CW, Chen JT, Galadima H, Eschbach K. The role of neighborhood characteristics in late stage melanoma diagnosis among hispanic men in california, Texas, and Florida, 1996-2012. *J Cancer Epidemio*l 2017;2017:8418904. 10.1155/2017/8418904 10.1155/2017/8418904 PMC5494113 28702054

[140] Kim MH, Schwartz GL, White JS, et al. School racial segregation and long-term cardiovascular health among Black adults in the US: A quasi-experimental study. *PLoS Med* 2022;19(6):e1004031. 10.1371/journal.pmed.1004031. 10.1371/journal.pmed.1004031 PMC9258802 35727819

[141] Moody HA, Darden JT, Pigozzi BW. The relationship of neighborhood socioeconomic differences and racial residential segregation to childhood blood lead levels in metropolitan detroit. *J Urban Health* 2016;93(5):820–39. 10.1007/s11524-016-0071-8. 10.1007/s11524-016-0071-8 PMC5052146 27538746

[142] Murosko D, Passerella M, Lorch S. Racial segregation and intraventricular hemorrhage in preterm infants. *Pediatrics* 2020;145(6). 10.1542/peds.2019-1508. 10.1542/peds.2019-1508 PMC11864279 32381625

[143] Poulson MR, Beaulieu-Jones BR, Kenzik KM, et al. Residential racial segregation and disparities in breast cancer presentation, treatment, and survival. *Ann Surg* 2021;273(1):3–9. 10.1097/sla.0000000000004451. 10.1097/SLA.0000000000004451 32889878

[144] Poulson MR, Helrich SA, Kenzik KM, Dechert TA, Sachs TE, Katz MH. The impact of racial residential segregation on prostate cancer diagnosis and treatment. *BJU Int* 2021;127(6):636–44. 10.1111/bju.15293. 10.1111/bju.15293 33166036

[145] Sathyanarayanan S, Brooks AJ, Hagen SE, Edington DW. Multilevel analysis of the physical health perception of employees: community and individual factors. *Am J Health Promot* 2012;26(5):e126–36. 10.4278/ajhp.110316-QUAL-120. 10.4278/ajhp.110316-QUAL-120 22548431

[146] Siegel M, Sherman R, Li C, Knopov A. The Relationship between Racial Residential Segregation and Black-White Disparities in Fatal Police Shootings at the City Level, 2013-2017. J Natl Med Assoc 2019;111(6):580–87. 10.1016/j.jnma.2019.06.003. 10.1016/j.jnma.2019.06.003 31256868

[147] Kehm RD, Misra DP, Slaughter-Acey JC, Osypuk TL. Measuring the effect of neighborhood racial segregation on fetal growth. *West J Nurs Res* 2022;44(1):5–14. 10.1177/01939459211037060. 10.1177/01939459211037060 PMC9867910 34378455

[148] Mancera N, Do DP, Griepentrog GJ, Esmaili N. Assault-related orbital trauma at an urban level I trauma center: racial segregation and other neighborhood-level social determinants. *Ophthalmic Plast Reconstr Surg* 2022. 10.1097/iop.0000000000002286. 10.1097/IOP.0000000000002286 36190913

[149] Tuliani TA, Shenoy M, Parikh M, Jutzy K, Hilliard A. Impact of area deprivation index on coronary stent utilization in a medicare nationwide cohort. Popul Health Manag 2017;20(4):329–34. 10.1089/pop.2016.0086. 10.1089/pop.2016.0086 28106520

