# Supplementary material for: Measuring Spatial Social Polarization in Public Health Research: A Scoping Review of Methods and Applications
Source: J Urban Health. 2025 Mar 10;102(2):213–39. doi: 10.1007/s11524-024-00957-6 (PMC12031708; doi:10.1007/s11524-024-00957-6)
Supplement: Supplementary file 1 — Supplementary file1 (DOCX 69 KB) [file 11524_2024_957_MOESM1_ESM.docx]

**A. Supplementary Materials**

**A1. Composite Indices**

In addition to the 18 SSP measures that were included in our review, we identified 5 composite indices across the socioeconomic status domain: *Area Deprivation Indices, Material Deprivation Indices, Social Deprivation Indices, Socioeconomic Status Indices, and Neighborhood Problems Scores*. Supplementary Table A1 describes each of the 5 composite indices, and provides background information, reference details, a count of the underlying items used to derive the measure (i.e., social factors, income factors, housing factors, occupation factors, education factors, and environmental factors), and notes on the consistency of underlying factors.

These composite indices include combinations of sociodemographic factors (SOC), income factors (INC), housing factors (HOU), occupational factors (OCC), educational factors (EDU), and/or environmental factors (ENV). Considering the heterogeneity in the underlying factors used in the composite indices, these indices were classified according to the methods used to derive each composite index. The most commonly employed composite indices were: Socioeconomic Status Indices (n=9), Area Deprivation Indices (n=8), and Material Deprivation Indices (n=8).

*Area Deprivation Indices* use socioeconomic variables, typically from census data, to provide an absolute measure of deprivation for a given area. In some cases, the underlying factors used to construct Area Deprivation Indices may be standardized, and in other cases these indices rely on dimensionality reduction techniques like Principal Component Analysis to guide the selection of underlying factors. A majority of Area Deprivation Indices include sociodemographic factors, income factors, housing factors, occupational factors, and educational factors [1-5], with few studies including environmental factors [6-8]. The Material and Social Deprivation Indices are similar to Area Deprivation Indices, in that they aim to measure different components of deprivation. However, both the Material Deprivation Indices and the Social Deprivation Indices are standardized in terms of the underlying factors included in the indices; these indices are estimated and made available by Statistics Canada [9].

The *Material Deprivation Index* is one of two deprivation indices developed by Pampalon et al. [9] to measure the material components of socioeconomic conditions among geographic units in Canada, it includes area-level measures for 3 socioeconomic indicators: income, education, and employment. The Material Deprivation Indices have been consistently employed in the literature [10-15], with one exception [16]. The *Social Deprivation Index* is the second of two deprivation indices developed by Pampalon et al. [9] to measure the social components of socioeconomic conditions among Canadian geographic units. Social Deprivation Indices synthesize area-level measures for 3 socioeconomic indicators: living alone, being separated/divorced/widowed, and being a single parent family. The Social Deprivation Index has been less routinely used [11, 14], and exhibits greater heterogeneity in the underlying factors used to derive the index [12, 17, 18]. Collectively, these indices allow researchers to assess the socioeconomic deprivation of a specific geographic unit.

We identified 9 articles that utilized *Socioeconomic Status Indices*; relative measures of socioeconomic status based on several indicators from US census data. While the methods used to construct Socioeconomic Status Indices are analogous to those of Area Deprivation Indices, the former differs from the latter, as Socioeconomic Status Indices indicate a unit’s relative socioeconomic position and not just deprivation. In terms of the items used (e.g., underlying factors) to derive these indices, studies consistently included sociodemographic factors, income factors, housing factors, occupational factors, and educational factors, with no studies including environmental factors [19-27]. Last, we identified 2 studies that used Neighborhood Problems Scores [28, 29]. *Neighborhood Problems Scores* are indices similar to Socioeconomic Status Indices, except these indices are designed to subjectively measure individual perceptions of an areas built and social environments. Neighborhood Problems Scores typically involve *de novo* questionnaire data and vary depending on the subjectively measure individual perceptions of an areas built and social environments that are included in the index. While there exist differences in how Neighborhood Problems Scores are operationalized, the studies identified by our review consistently featured several indicators for ENV factors [28, 29]. Together, these indices represent various methods of delineating the relative socioeconomic position of a geographic unit.

Considering differences in the underlying factors that were used to derive the composite indices, these measures were classified accordingly and examined in terms of the factors used to construct the index. Distinguishing between SSP measures and composite indices draws attention to the various types of measures identified by our review, and the respective methods involved in metric construction. In general, methods for composite indices relied on variable selection, guided by either standardization, or principal component analysis. Some composite indices, like the Social Deprivation Index and the Material Deprivation Index, were for the most part consistently derived. These indices are standardized based on Canadian census data via Statistics Canada [9]. Unlike Canada, the US lacks centralized guidance, which is evident in the heterogeneity of items used to derive indices like the Socioeconomic Status Indices. While there are attempts to standardize composite indices in the US, such as using the Social Vulnerability Index [30], our search failed to identify any standardized composite indices for use in US populations. In terms of composite indices, researchers should make every reasonable effort to use a standardized measure - if available, and if not-available, at a minimum disclose the details of the underlying factors that were used to create the index. Transparency in the types of measures used, and the methods used to construct measures ensures reproducibility and accountability, both of which are crucial to the advancement of public health research and practice.

| **Supplementary Table A1** Composite Indices | | | | | | | | | | |
| --- | --- | --- | --- | --- | --- | --- | --- | --- | --- | --- |
| **Composite Index** | **Background/Methods** | **Ref.**  **Num.** | **Country** | **Items Used*** (n) | | | | | | **Notes on**  **Consistency** |
|  |  |  |  | *SOC* | *INC* | *HOU* | *OCC* | *EDU* | *ENV* |  |
| *1* |  |  |  |  |  |  |  |  |  |  |
| *Material Deprivation Indices* | The Material Deprivation Index is one of two deprivation indices developed by Pampalon et al. 2000 [9] to **measure the material components of socioeconomic conditions** among geographic areas in Canada. The Material Deprivation index synthesizes area-level measures for 3 socioeconomic indicators: income, education, and employment. The Pampalon deprivation indices are consistently estimated and made available by the Canadian Census (Statistics Canada). The index is constructed using Principal Component Analyses. | [10] | CANADA | 0 | 1 | 0 | 1 | 1 | 0 | The Material Deprivation Index developed by Pampalon et al. 2000 [9] are consistently employed in the literature, despite heterogeneity in the operationalization of underlying items. This index provides an interesting contrast to the “Area Deprivation Indices”. |
|  |  | [11] | CANADA | 0 | 1 | 0 | 1 | 1 | 0 |  |
|  |  | [12] | CANADA | 0 | 1 | 0 | 1 | 1 | 0 |  |
|  |  | [13] | CANADA | 0 | 1 | 0 | 1 | 1 | 0 |  |
|  |  | [14] | CANADA | 0 | 1 | 0 | 1 | 1 | 0 |  |
|  |  | [15] | CANADA | 0 | 1 | 0 | 1 | 1 | 0 |  |
|  |  | [16] | CANADA | 0 | 1 | 0 | 1 | 1 | 0 |  |
|  |  | [18] | CANADA | 2 | 1 | 0 | 0 | 1 | 1 |  |
| *Social Deprivation Indices*  *Social Deprivation Indices*  *(cont’d)* | The Social Deprivation Index is one of two deprivation indices developed by Pampalon et al. 2000 [9] to **measure the social components of socioeconomic conditions** among geographic areas in Canada. The Social Deprivation index synthesizes area-level measures for 3 socioeconomic indicators: living alone, being separated/divorced/widowed, and being a single parent family. The Pampalon deprivation indices are consistently estimated and made available by the Canadian Census (Statistics Canada). Principal Component Analysis is used to generate factor scores for the indicators, which are then ranked accordingly. | [11] | CANADA | 3 | 0 | 0 | 0 | 0 | 0 | The Social Deprivation Index is consistently described in the body of evidence, however, there exist differences in the underlying items used. Compared to the “Material Deprivation Index”, the Social Deprivation Index is less commonly employed in recent public health literature. |
|  |  | [14] | CANADA | 3 | 0 | 0 | 0 | 0 | 0 |  |
|  |  | [18] | CANADA | 2 | 0 | 1 | 0 | 0 | 1 |  |
|  |  | [17] | FRANCE | 0 | 1 | 0 | 2 | 1 | 0 |  |
|  |  | [12] | CANADA | 1 | 0 | 1 | 1 | 1 | 0 |  |
| *Neighborhood Problems Scores* | Neighborhood Problems Scores are indices designed to **subjectively measure individual perceptions** of a neighborhood’s built and social environment(s). These indices are often derived from de novo questionnaire data, with variable selection informed by existing evidence. Questionnaires are typically on a Likert scale, of which items are summed to derive an overall score; interpretation depends on how scale is operationalized. | [29] | US | 0 | 0 | 0 | 0 | 0 | 6 | Neighborhood Problems Scores are unique in that they are derived from de novo questionnaire data, which may leverage previously validated survey items, and result in the differential inclusion/exclusion of specific factors. Notably, these Neighborhood Problems Scores all featured ENV factors related to the physical and social aspects of neighborhood disinvestment and disorder including *lack of recreational facilities*, *poorly maintained sidewalks*, *traffic*, and *excessive noise.* |
|  |  | [28] | US | 3 | 3 | 0 | 1 | 2 | 8 |  |
| *Socioeconomic Status Indices*  *Socioeconomic Status Indices*  *(cont’d)* | Socioeconomic Status Indices are **relative measures of socioeconomic status**. These indices are composite measures that aim to capture different socio-economic contexts of neighborhoods and other geographic areas. These composite measures typically include several indicators from the US Census, which are subjected to Principal Component Analysis to guide factor selection. | [20] | ITALY | 3 | 1 | 1 | 1 | 1 | 0 | These indices are employed under several names including "summary neighborhood socioeconomic score", "modified Darden Kamel socioeconomic index" and "composite neighborhood socioeconomic status index". There are differences in which underlying items are included. Collectively, the discordances in naming conventions and the application of underlying items indicates low consistency across metrics. |
|  |  | [21] | US | 1 | 1 | 2 | 1 | 1 | 0 |  |
|  |  | [23] | US | 0 | 2 | 1 | 1 | 2 | 0 |  |
|  |  | [22] | US | 1 | 3 | 0 | 1 | 1 | 0 |  |
|  |  | [26] | US | 1 | 2 | 0 | 2 | 0 | 0 |  |
|  |  | [19] | US | 0 | 1 | 2 | 1 | 1 | 0 |  |
|  |  | [25] | US | 0 | 1 | 1 | 1 | 1 | 0 |  |
|  |  | [24] | US | 1 | 2 | 0 | 1 | 1 | 0 |  |
|  |  | [27] | US | 1 | 2 | 0 | 1 | 0 | 0 |  |
| *Area Deprivation Indices* | Area Deprivation Indices leverage socioeconomic census data to provide an **absolute measure of area deprivation**; higher index values indicate greater deprivation for a given area. Variable selection and index derivation is typically guided by Principal Component Analyses. | [8] | US | 0 | 3 | 1 | 0 | 1 | 1 | Given the lack of a gold-standard in the US, there is heterogeneity in the factors included in Area Deprivation Indices; a majority of indices included SOC, INC, HOU, OCC, and EDU factors, with only a handful of indices including ENV factors. INC & HOU factors such as *family income*, *home value*, and *poverty* were among the most frequent included factors. |
|  |  | [7] | US | 3 | 4 | 4 | 2 | 2 | 2 |  |
|  |  | [6] | US | 3 | 4 | 4 | 2 | 2 | 2 |  |
|  |  | [3] | US | 1 | 2 | 0 | 1 | 1 | 0 |  |
|  |  | [5] | CANADA | 1 | 1 | 1 | 2 | 2 | 0 |  |
|  |  | [1] | US | 2 | 4 | 1 | 2 | 2 | 0 |  |
|  |  | [2] | US | 2 | 3 | 2 | 1 | 1 | 0 |  |
|  |  | [4] | US | 1 | 2 | 1 | 2 | 1 | 0 |  |
| Footnote: * denotes the number of factors used across each domain: sociodemographic (SOC), income (INC), occupational (OCC), education (EDU), and environmental (ENV); darker shades of gray indicate a greater number of factors and vice-versa. | | | | | | | | | | |

**References for Supplementary Materials**

1. Sathyanarayanan S, Brooks AJ, Hagen SE, Edington DW. Multilevel analysis of the physical health perception of employees: community and individual factors. American journal of health promotion : AJHP 2012;**26**(5):e126-36 doi: 10.4278/ajhp.110316-QUAL-120.

2. Mason SM, Messer LC, Laraia BA, Mendola P. Segregation and preterm birth: the effects of neighborhood racial composition in North Carolina. Health Place 2009;**15**(1):1-9 doi: 10.1016/j.healthplace.2008.01.007.

3. Kehm RD, Misra DP, Slaughter-Acey JC, Osypuk TL. Measuring the Effect of Neighborhood Racial Segregation on Fetal Growth. West J Nurs Res 2022;**44**(1):5-14 doi: 10.1177/01939459211037060.

4. Hruska B, Pacella-LaBarbara ML, Castro IE, George RL, Delahanty DL. Incorporating community-level risk factors into traumatic stress research: Adopting a public health lens. Journal of anxiety disorders 2022;**86**:102529 doi: 10.1016/j.janxdis.2022.102529.

5. Bell N, Schuurman N, Hameed SM. A multilevel analysis of the socio-spatial pattern of assault injuries in greater Vancouver, British Columbia. Canadian journal of public health = Revue canadienne de sante publique 2009;**100**(1):73-7 doi: 10.1007/bf03405498.

6. Tuliani TA, Shenoy M, Parikh M, Jutzy K, Hilliard A. Impact of Area Deprivation Index on Coronary Stent Utilization in a Medicare Nationwide Cohort. Popul Health Manag 2017;**20**(4):329-34 doi: 10.1089/pop.2016.0086.

7. Eick SM, Cushing L, Goin DE, et al. Neighborhood conditions and birth outcomes: Understanding the role of perceived and extrinsic measures of neighborhood quality. Environ Epidemiol 2022;**6**(5):e224 doi: 10.1097/ee9.0000000000000224.

8. Burris HH, Mullin AM, Dhudasia MB, et al. Neighborhood Characteristics and Racial Disparities in Severe Acute Respiratory Syndrome Coronavirus 2 (SARS-CoV-2) Seropositivity in Pregnancy. Obstetrics and gynecology 2022;**139**(6):1018-26 doi: 10.1097/aog.0000000000004791.

9. Pampalon R, Raymond G. A deprivation index for health and welfare planning in Quebec. Chronic Dis Can 2000;**21**(3):104-13.

10. Abda A, Del Giorgio F, Gauvin L, Autmizguine J, Kakkar F, Drouin O. Association between area-level material deprivation and incidence of hospitalization among children with SARS-CoV-2 in Montreal. Paediatr Child Health 2022;**27**(Suppl 1):S27-s32 doi: 10.1093/pch/pxab106.

11. Blais C, Hamel D, Rinfret S. Impact of socioeconomic deprivation and area of residence on access to coronary revascularization and mortality after a first acute myocardial infarction in Québec. The Canadian journal of cardiology 2012;**28**(2):169-77 doi: 10.1016/j.cjca.2011.10.009.

12. Gupta N, Crouse DL, Miah P, Takaro T. The role of neighbourhood environments in hospitalization risk for diabetes and related conditions: A population-based cohort analysis by remoteness and deprivation indices. Health Rep 2022;**33**(12):3-13 doi: 10.25318/82-003-x202201200001-eng.

13. Meshefedjian GA, Ouimet MJ, Frigault LR, Leaune V, Ait Kaci Azzou S, Simoneau M. Association of Material Deprivation Status, Access to Health Care Services, and Lifestyle With Screening and Prevention of Disease, Montreal, Canada, 2012. Preventing chronic disease 2016;**13**:E137 doi: 10.5888/pcd13.160157.

14. Saint-Jacques N, Dewar R, Cui Y, Parker L, Dummer TJ. Premature mortality due to social and material deprivation in Nova Scotia, Canada. Int J Equity Health 2014;**13**(1):94 doi: 10.1186/s12939-014-0094-2.

15. Samadoulougou S, Letarte L, Lebel A. Association between Neighbourhood Deprivation Trajectories and Self-Perceived Health: Analysis of a Linked Survey and Health Administrative Data. Int J Environ Res Public Health 2022;**20**(1) doi: 10.3390/ijerph20010486.

16. Letarte L, Samadoulougou S, McKay R, Quesnel-Vallée A, Waygood EOD, Lebel A. Neighborhood deprivation and obesity: Sex-specific effects of cross-sectional, cumulative and residential trajectory indicators. Social science & medicine (1982) 2022;**306**:115049 doi: 10.1016/j.socscimed.2022.115049.

17. Scherpereel A, Durand-Zaleski I, Cotté FE, et al. Access to innovative drugs for metastatic lung cancer treatment in a French nationwide cohort: the TERRITOIRE study. BMC Cancer 2018;**18**(1):1013 doi: 10.1186/s12885-018-4958-5.

18. Zandy M, Zhang LR, Kao D, et al. Area-based socioeconomic disparities in mortality due to unintentional injury and youth suicide in British Columbia, 2009-2013. Health Promot Chronic Dis Prev Can 2019;**39**(2):35-44 doi: 10.24095/hpcdp.39.2.01.

19. Borrell LN, Kiefe CI, Diez-Roux AV, Williams DR, Gordon-Larsen P. Racial discrimination, racial/ethnic segregation, and health behaviors in the CARDIA study. Ethnicity & health 2013;**18**(3):227-43 doi: 10.1080/13557858.2012.713092.

20. Camilloni L, Farchi S, Chini F, Giorgi Rossi P, Borgia P, Guasticchi G. How socioeconomic status influences road traffic injuries and home injuries in Rome. Int J Inj Contr Saf Promot 2013;**20**(2):134-43 doi: 10.1080/17457300.2012.692695.

21. Cozier YC, Yu J, Coogan PF, Bethea TN, Rosenberg L, Palmer JR. Racism, segregation, and risk of obesity in the Black Women's Health Study. Am J Epidemiol 2014;**179**(7):875-83 doi: 10.1093/aje/kwu004.

22. Do DP, Watkins DC, Hiermeyer M, Finch BK. The relationship between height and neighborhood context across racial/ethnic groups: a multi-level analysis of the 1999-2004 U.S. National Health and Nutrition Examination Survey. Economics and human biology 2013;**11**(1):30-41 doi: 10.1016/j.ehb.2012.01.003.

23. Jiang L, Chang J, Beals J, Bullock A, Manson SM. Neighborhood characteristics and lifestyle intervention outcomes: Results from the Special Diabetes Program for Indians. Preventive medicine 2018;**111**:216-24 doi: 10.1016/j.ypmed.2018.03.009.

24. Khanijahani A, Tomassoni L. Socioeconomic and Racial Segregation and COVID-19: Concentrated Disadvantage and Black Concentration in Association with COVID-19 Deaths in the USA. J Racial Ethn Health Disparities 2022;**9**(1):367-75 doi: 10.1007/s40615-021-00965-1.

25. Masi CM, Hawkley LC, Piotrowski ZH, Pickett KE. Neighborhood economic disadvantage, violent crime, group density, and pregnancy outcomes in a diverse, urban population. Social science & medicine (1982) 2007;**65**(12):2440-57 doi: 10.1016/j.socscimed.2007.07.014.

26. Moody HA, Darden JT, Pigozzi BW. The Relationship of Neighborhood Socioeconomic Differences and Racial Residential Segregation to Childhood Blood Lead Levels in Metropolitan Detroit. Journal of urban health : bulletin of the New York Academy of Medicine 2016;**93**(5):820-39 doi: 10.1007/s11524-016-0071-8.

27. Pinchak NP, Browning CR, Calder CA, Boettner B. Racial Inequalities in Adolescents' Exposure to Racial and Socioeconomic Segregation, Collective Efficacy, and Violence. Demography 2022;**59**(5):1763-89 doi: 10.1215/00703370-10210688.

28. Samuel LJ, Thorpe RJ, Jr., Bower KM, LaVeist TA. Community Characteristics are Associated with Blood Pressure Levels in a Racially Integrated Community. Journal of urban health : bulletin of the New York Academy of Medicine 2015;**92**(3):403-14 doi: 10.1007/s11524-015-9936-5.

29. Duncan DT, Sutton MY, Park SH, et al. Associations Between Neighborhood Problems and Sexual Behaviors Among Black Men Who Have Sex with Men in the Deep South: The MARI Study. Arch Sex Behav 2020;**49**(1):185-93 doi: 10.1007/s10508-019-01619-4.

30. Fletcher KM, Espey J, Grossman MK, et al. Social vulnerability and county stay-at-home behavior during COVID-19 stay-at-home orders, United States, April 7–April 20, 2020. Annals of epidemiology 2021;**64**:76-82.
